# Genetic factors affecting Fusarium head blight resistance improvement from introgression of exotic Sumai 3 alleles (including *Fhb1*, *Fhb2*, and *Fhb5*) in hard red spring wheat

**DOI:** 10.1186/s12870-019-1782-2

**Published:** 2019-05-03

**Authors:** Gurcharn Singh Brar, Anita L. Brûlé-Babel, Yuefeng Ruan, Maria Antonia Henriquez, Curtis Jerry Pozniak, Hadley Randal Kutcher, Pierre Jan Hucl

**Affiliations:** 1 Crop Development Centre/Department of Plant Science, University of Saskatchewan, 51 Campus Dr, Saskatoon, SK S7N 5A8 Canada; 2 Department of Plant Science, University of Manitoba, 66 Dafoe Road, Winnipeg, MB R3T 2N2 Canada; 3 Present address: Agriculture and Agri-Food Canada, Swift Current Research and Development Centre, 1 Airport Road, Swift Current, SK S9H 3X2 Canada; 4 Agriculture and Agri-Food Canada, Morden Research and Development Centre, 101 Route 100, Morden, MB R6M 1Y5 Canada

**Keywords:** Cereals, Epistatic interactions, Fusarium head blight, *Fhb1*, *Fhb2*, *Fhb5*, Scab, Sumai 3, Wheat

## Abstract

**Background:**

Fusarium head blight resistance genes, *Fhb1* (for Type-II resistance), *Fhb2* (Type-II), and *Fhb5* (Type-I plus some Type-II), which originate from Sumai 3, are among the most important that confer resistance in hexaploid wheat. Near-isogenic lines (NILs), in the CDC Alsask (susceptible; *n* = 32) and CDC Go (moderately susceptible; *n* = 38) backgrounds, carrying these genes in all possible combinations were developed using flanking microsatellite markers and evaluated for their response to FHB and deoxynivalenol (DON) accumulation in eight environments. NILs were haplotyped with wheat 90 K iSelect assay to elucidate the genomic composition and confirm alleles’ presence. Other than evaluating the effects of three major genes in common genetic background, the study elucidated the epistatic gene interactions as they influence FHB measurements; identified loci other than *Fhb1*, *Fhb2*, and *Fhb5*, in both recurrent and donor parents and examined annotated proteins in gene intervals.

**Results:**

Genotyping using 81,857 single nucleotide polymorphism (SNP) markers revealed polymorphism on all chromosomes and that the NILs carried < 3% of alleles from the resistant donor. Significant improvement in field resistance (Type-I + Type-II) resulted only among the CDC Alsask NILs, not the CDC Go NILs. The phenotypic response of NILs carrying combinations of Sumai 3 derived genes suggested non-additive responses and *Fhb5* was as good as *Fhb1* in conferring field resistance in both populations. In addition to *Fhb1*, *Fhb2*, and *Fhb5,* four to five resistance improving alleles in both populations were identified and three of five in CDC Go were contributed by the susceptible parent. The introgressed chromosome regions carried genes encoding disease resistance proteins, protein kinases, nucleotide-binding and leucine rich repeats’ domains. Complex epistatic gene-gene interactions among marker loci (including *Fhb1*, *Fhb2*, *Fhb5*) explained > 20% of the phenotypic variation in FHB measurements.

**Conclusions:**

Immediate Sumai 3 derivatives carry a number of resistance improving minor effect alleles, other than *Fhb1*, *Fhb2*, *Fhb5*. Results verified that marker-assisted selection is possible for the introgression of exotic FHB resistance genes, however, the genetic background of the recipient line and epistatic interactions can have a strong influence on expression and penetrance of any given gene.

**Electronic supplementary material:**

The online version of this article (10.1186/s12870-019-1782-2) contains supplementary material, which is available to authorized users.

## Background

Wheat (*Triticum* spp. L.) is one of the most important field crops worldwide as it serves as staple food for a large proportion of the global population. Wheat production is challenged by several constraints and Fusarium head blight is one of the major biotic limitations. There are several *Fusarium* spp. that cause head blight or scab; *Fusarium graimnearum* Schwabe (syn. *Gibberella zeae* Schw. [Petch]) is the main culprit in North America, and it is also hosted by maize (*Zea mays* L.) and barley (*Hordeum vulgare* L.) [1, 2]. Direct yield loss from the disease is due to shrivelled grain with lower test weight or even failure of seed formation. Loss in marketability from mycotoxins accumulation is an even bigger concern from an international trade perspective. The accumulation of harmful mycotoxins, particularly deoxynivalenol (DON) and its acetylated forms (3-ADON and 15-ADON), may render the grain unsuitable for food or feed. The majority of wheat growing countries have defined certain threshold limits for the presence of DON in the grain to be able to export or import across international boundaries and many beverage and food industries have self-imposed even greater restrictions [1].

An integrated approach for FHB management is imperative and genetic resistance is an integral part of such disease management approach. Resistance to FHB in wheat is inherited quantitatively and strongly influenced by the environment [3]. Genetic studies in wheat have identified many useful loci for improvement in complex traits, such as FHB; unfortunately, many of them remain un- or under-utilized in plant breeding programs mainly because of the complex nature of resistance [3]. In spite of a tremendous amount of FHB resistance breeding efforts, genetic gain has been moderate [4]. Efficient introgression of QTL associated with FHB resistance into elite germplasm requires the use of linked genetic markers to facilitate marker-assisted selection (MAS); however, the linkage phase between the marker(s) and the QTL cannot always be inferred among genetic backgrounds, unless strong linkage disequilibrium exists [5]. Of more than 100 QTL identified for resistance to FHB, only seven have been formally designated as Mendelized genes: *Fhb1* derived from Sumai 3 [6], *Fhb2* from Sumai 3 [7], *Fhb3* from *Leymus racemosus* [8], *Fhb4* from Wangshuibai [9], *Fhb5* from Wangshuibai and Sumai 3 [10], *Fhb6* from *Elymus tsukushiensis* [11], and *Fhb7* from *Thinopyrum ponticum* [12]. Based on host response, the expression of resistance is classified into five different types: Type-I (resistance to initial pathogen infection), Type-II (resistance to fungal spread in the spike), Type-III (resistance to toxin accumulation or the ability to degrade the mycotoxins), Type-IV (resistance to kernel infection), and Type-V (tolerance to yield loss) [13–15]. Type-I and Type-II resistance are more widely exploited and Type-III resistance has gained importance as it is important to maintain grain end-use quality. All types of resistance are generally moderately to well correlated.

The discovery of promising QTL is a preliminary step in a MAS program, but validation of such loci in multiple genetic backgrounds and environments is equally important [16]. The actual effect of the QTL, usually identified from bi-parental populations such as recombinant inbred lines or double haploid lines, is dependent on the alleles and allelic frequencies present at the locus, as well as epistatic interactions among QTL and other genes, which are usually over-estimated in the original mapping population [5]. Near-isogenic lines (NILs) are advantageous for studying phenotypic effects attributable to a specific QTL or gene as the genetic background is fixed, which in turn maintains morphological and phenological traits of the plants that might influence the trait under study [17]. NILs are particularly attractive to breeders for traits that are introduced from exotic parents or wide crosses as they allow confirmation of allelic effects on traits of interest. Additionally, by fixing the genetic background, NILs serve as an ideal source for fine-mapping, gene expression profiling, and hypothesis-driven biological experimentation [18].

Canadian wheat growers have witnessed several FHB epidemics in the last two decades, particularly in eastern Canada and the province of Manitoba in western Canada [2, 19]. However, in last 10–15 years, FHB epidemics in Saskatchewan are not uncommon; attributable to the increasing proportion of the more aggressive 3-ADON chemotype in the *F. graminearum* population [19, 20]. These epidemics spurred research to improve genetic resistance and management options for FHB. The majority of the resistance genes currently available originate from Asian or Brazilian wheats; however, breeders in North America are reluctant to use exotic sources in their programs due to linkage drag (for example, shattering and susceptibility of Sumai 3 to other pathogens). In an effort to introgress resistance into Canadian hard red spring wheat, the bread wheat breeding program at the Crop Development Centre (CDC) utilized 04GC0139 (pedigree: ND2710/RL4851//BW278; carrying *Fhb1*, *Fhb2*, and *Fhb5*), a derivative of Sumai 3, to cross with CDC wheat cultivars. The current project used NILs in CDC Alsask and CDC Go backgrounds to study the effects of these three major genes and their combinations: *Fhb1*, *Fhb2*, and *Fhb5*. The effects of introgressing *Fhb1* and *Fhb5* on disease resistance are reported in previously published studies from North America [18, 21, 22], Europe [23, 24], and China [25]. The majority of these studies used RILs/NILs from F_2_ derived inbreds through enforced inbreeding or doubled haploid lines and only Salameh et al. [24] and Xue et al. [25] used BC_2_ or BC_3_ derived NILs; thus, a greater proportion of the resistant donor alleles was expected in these studies, which influenced the overall phenotypic expression of the lines. Additionally, these studies utilized only microsatellite (SSR) markers spanning a large physical interval, unlike modern KASP/SNP markers associated with a single gene region. Therefore, to precisely quantify the effect of major FHB resistance genes or QTL, it is imperative to reduce the proportion of other alleles as much as possible. At the same time, it is practically impossible (with repeated backcrossing or other classical breeding approaches) to introgress only genes of interest in any given genetic background, thus, one should account for the effect of other alleles and their interaction with genes of interest to influence phenotypic expression.

The specific objectives of the current study were: (i) to examine the phenotypic effect on FHB resistance from the introgression of Sumai 3-derived genes in multiple elite backgrounds with differential susceptibility, (ii) to determine the allelic proportion in two backgrounds, derived from the resistant donor parent, and (iii) to examine single marker-effect and marker-marker interactions for all polymorphic markers among NIL entries. Here, we report the effect of *Fhb1*, *Fhb2*, and *Fhb5* genes on FHB severity and DON accumulation in two hard red spring wheat cultivars, one that was moderately susceptible (MS) and the other susceptible (S) to the disease. The genomic composition of the NILs was thoroughly analyzed for allelic effects and the proportions of alleles from each parent. The study essentially characterized the complexity of the trait through gene-gene interactions and identified loci other than *Fhb1*, *Fhb2*, and *Fhb5* that contribute to improved FHB resistance.

## Results

### Marker analyses

Molecular characterization of the NILs in both populations using gene-specific microsatellite or SNP markers assisted in their classification into individual gene and gene combination classes. Additional genotypic data were generated with the 90K iSelect wheat assay [26] to determine the genomic composition and the haplotype structure of the NILs compared to their recurrent parents. The SNP markers were assigned to chromosomes using the reference sequence assembly of Chinese Spring RefSeq ver. 1 (International Wheat Genome Sequencing Consortium, https://wheat-urgi.versailles.inra.fr/Seq-Repository/Assemblies). Polymorphisms among NILs were present on all 21 chromosomes in both NIL populations (Additional file 1: Table S1, Figure S1-S3). A total of 10,535 SNPs were polymorphic among parents in the CDC Alsask population and 8686 SNPs in the CDC Go population; however, only 3667 and 1454 were polymorphic among the NILs (Additional file 1: Figure S1). Of the polymorphic SNPs among the NILs, most of the markers were located on Chromosomes 3B (452), 5A (444), 5B (341), and 6B (510) in CDC Alsask, and on 1A (127), 2A (167), 3B (83), 5A (226), and 7A (188) in CDC Go. The chromosomes 3B, 5A, 6B carrying *Fhb1*, *Fhb5*, and *Fhb2* were anchored with 38% of the total polymorphic SNPs in CDC Alsask and 26% in CDC Go.

**Fig. 1:**
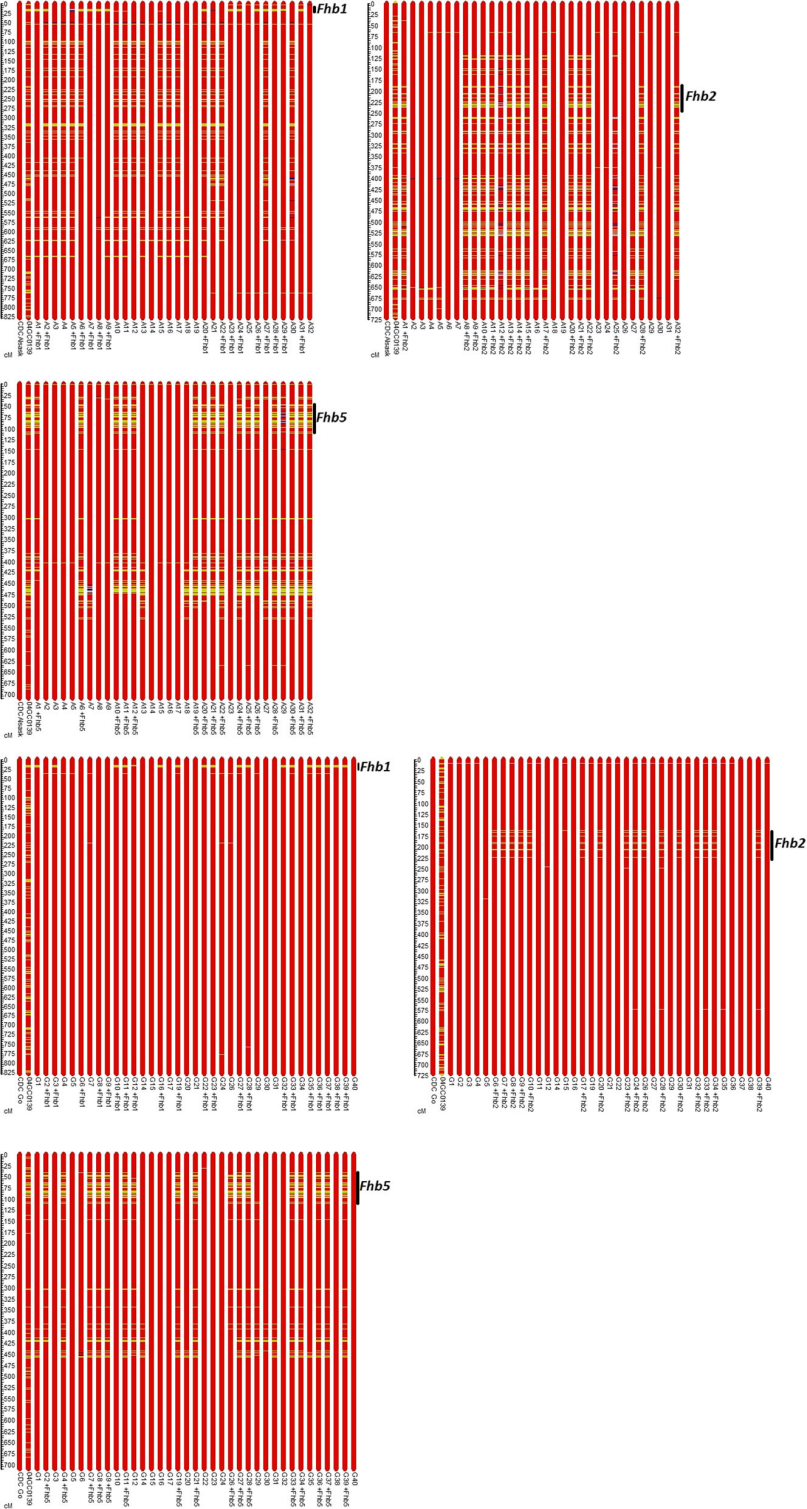
Physical positions of *Fhb1*, *Fhb2*, *Fhb5* in CDC Go and CDC Alsask near-isogenic lines (NILs). Graphical presentation of physical position of introgressed segments on chromosomes 3B (carrying *Fhb1*), 6B (carrying *Fhb2*), 5A (carrying *Fhb5*) from 04GC0139 (resistance donor parent, yellow segments) into CDC Alsask (upper panel) and CDC Go (lower panel) (red segments) near-isogenic lines. The scale bar on left hand side indicates physical position (Mb) and the black bar on the right indicates haplotype segment carrying *Fhb1*, *Fhb2* or *Fhb5* gene. Each bar represents a genotype. The grey and blue segments indicate unknown and heterozygous alleles, respectively

With the help of previously published studies or the use of a consensus map and physical location of the SNPs, the markers on Chromosomes 3BS, 5AS, and 6BS from the 90K assay were identified and used to define haplotype segments carrying *Fhb1*, *Fhb2*, and *Fhb5* in both populations (Fig. 1) [27–29]; Ron Knox, unpublished data]. The classification of the NILs into gene classes using microsatellite markers was in agreement with SNP markers for all three genes in both populations with the exception of *Fhb1* in two NIL entries (Go2 and Go6) in the CDC Go background. The two inconsistent NIL entries for presence/absence of *Fhb1* were classified using the functional gene-specific, pore-forming toxin (PFT) protein marker [30]. The CDC Alsask NILs carried more alleles (difference in recombination rate, a function of genetic background) from the donor parent as compared to the CDC Go NILs (Fig. 1; Additional file 1 Table S1, Figures S1-S3). The genomes of the CDC Alsask NILs carried up to 2.7% of the resistant donor’s alleles and the CDC Go NILs 0.9% (Additional file 1 Table S1). On Chromosomes 3B (carrying *Fhb1*), 5A (carrying *Fhb5*), and 6B (carrying *Fhb2*), the CDC Alsask NILs had up to 9.9, 11.6, and 12.6% of the resistant donor parent alleles, respectively, while the CDC Go NILs had 2.2, 6.7, and 1.7%.

### FHB evaluations and heritability estimates

In all environments, FHB inoculations were successful and there was sufficient disease pressure in all environments to discriminate NIL entries as indicated by longer environmental vectors in biplots which were proportional to the standard deviation in the phenotypic data (Additional file 1: Figure S4). Also, there was positive and significant correlations (*P* = 0.05) among most of the environments in both CDC Go and CDC Alsask populations, indicated by acute angles between environmental vectors. Two axes of the biplots explained ~ 56% of the phenotypic variation in both populations. For GH evaluations, *F*-values were significant for variation among gene classes in both NIL populations for GH14, GH21, and GH_AUDPC (Table 1). The *F*-values for entry nested within gene class were significant for GH14, GH21, and GH_AUDPC in the CDC Go population, but only for GH_AUDPC in the CDC Alsask population. The two *F. graminearum* chemotypes differed for GH14 and GH_AUDPC in both populations, whereas the interaction of chemotype by gene was significant only for GH_AUDPC in the CDC Alsask population. For field evaluations, *F*-values were significant only for gene classes for all FHB parameters in both populations. Insignificant *F*-values for entry nested within gene class for INC, SEV, IND, and DON indicated that the NIL entries within any given gene class behaved similarly. Broad-sense heritability (*H*^*2*^) estimates were high for GH14, GH21, GH_AUDPC, moderate for INC, SEV, IND and weak for DON. In both GH and field evaluations, the random effect of environment (site-year) was significant (*P* = 0.05; data not presented).

**Table 1:** Analysis of variance (*F*-values; across all environments) for near-isogenic lines (NILs) in CDC Go and CDC Alsask backgrounds, carrying all combinations of three Fusarium head blight (FHB) resistance genes: *Fhb1*, *Fhb2*, and *Fhb5*. Fixed effects of FHB resistance genes and entry (nested within gene) are provided for FHB severity assessed in the greenhouse at 14 days post inoculation (GH14), 21 days post inoculation (GH21), area under disease progress curve (GH_AUDPC), field incidence (FLD_INC), field severity (FLD_SEV), field FHB index (FLD_IND), deoxynivalenol (FLD_DON) accumulation, and broad-sense heritability (*H*^*2*^). For greenhouse data, the effect of chemotype (3ADON or 15ADON), and chemotype by gene interaction is also presented

Effect	df^a^	GH14	GH21	GH_AUDPC	FLD_INC	FLD_SEV	FLD_IND	FLD_DON
*CDC Alsask*								
Gene (G)	12	82.47***	364.28*	178.70***	9.60***	16.82***	13.25***	5.20***
Gene (entry)	29	2.29^ns^	3.42^ns^	3.90***	1.86^ns^	1.21^ns^	0.98^ns^	1.04^ns^
Chemotype (C)	1	11.38**	1.41^ns^	19.80***	–	–	–	–
C*G	12	0.99^ns^	1.01^ns^	2.17*	–	–	–	–
*H*^*2*^ (%)	–	87.5	92.4	90.6	77.3	52.3	59.6	21.4
*CDC Go*								
Gene (G)	12	19.71***	25.19***	25.60***	11.47***	9.74***	10.92***	7.39***
Gene (entry)	30	2.03**	1.65*	2.04**	1.07^ns^	1.03^ns^	0.95^ns^	1.13^ns^
Chemotype (C)	1	8.03*	3.02^ns^	7.38*	–	–	–	–
C*G	12	0.99^ns^	1.15^ns^	1.12^ns^	–	–	–	–
*H*^*2*^ (%)	–	80.9	90.4	86.3	58.0	56.3	45.0	15.4

Note: *, **, ***: significant at *P* < 0.05, *P* < 0.001, *P* < 0.0001, respectively; ns – not significant

^a^Degree of freedom

**Fig. 2:**
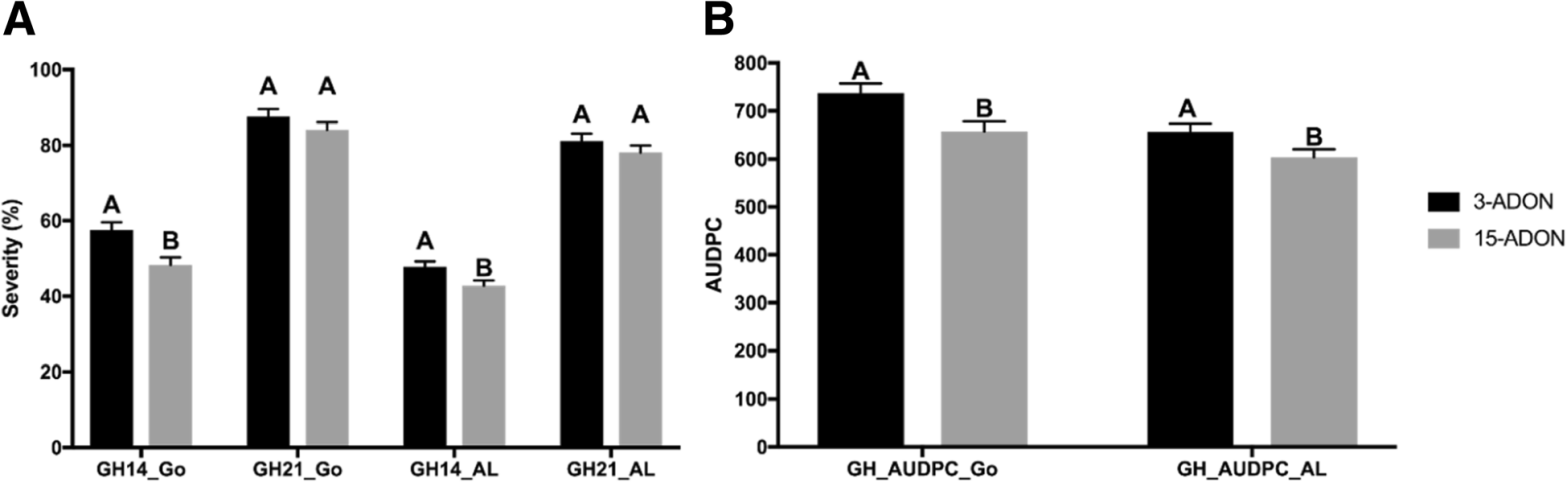
Greenhouse evaluation of near-isogenic lines (NILs) with 3-ADON and 15-ADON chemotypes of *Fusarium graminearum*. Fusarium head blight severity in CDC Go and CDC Alsask NILs following point inoculation with 3-ADON and 15-ADON chemotypes of *Fusarium graminearum* (50,000 macroconidia/ml) (**a**) in the greenhouse at 14 and 21 days post inoculation (dpi) (**b**) Area under disease progress curve (AUDPC) was calculated from three ratings: 7, 14, and 21 dpi. Bars with the same letter code are not statistically significantly different according to Fisher’s least significant differences at *P* = 0.05. The LSmeans were calculated from all NILs (excluding parents and checks) in each population

In the CDC Alsask population, for GH14, NILs carrying *Fhb1*, *Fhb5*, *Fhb2 + Fhb5* and *Fhb1 + Fhb2 + Fhb5* reduced FHB severity (SEV) as compared to the recurrent parent and the susceptible checks (Table 2). Other genes or gene combinations tended to lower the disease as compared to the recurrent parent and the susceptible checks, but differences were not statistically significant. For GH21 and GH_AUDPC, all genes and their combinations reduced disease compared to the recurrent parent and the susceptible check. For the CDC Go population, except for *Fhb2*, all other genes or their combinations reduced disease severity in GH14. For GH21, only NILs carrying all three genes reduced FHB severity and for GH_AUDPC, all genes classes except *Fhb1* and *Fhb2* had lower disease severity than the recurrent parent or the susceptible check. Pyramiding *Fhb1 + Fhb2 + Fhb5* in the CDC Alsask background reduced severity (relative to the recurrent parent) by 37% for GH14, 27% for GH21, and 39% for GH_AUDPC, and in the CDC Go background by 62, 33, and 49%, respectively. Although NILs carrying all three genes performed better than the intermediate/moderately resistant (MR) check, i.e. AC Barrie, the improvement was not comparable to the resistant check or the resistant donor parent. It is important to mention that the NILs that did not carry *Fhb1*, *Fhb2*, or *Fhb5* also reduced disease by 1–13% in both populations, and the combination of any two of these genes in the CDC Alsask population did not improve resistance as compared to NILs carrying the genes singly, which indicated significant gene interactions. The 3-ADON chemotype resulted in higher FHB severity, for GH14 and GH_AUDPC, as compared to the 15-ADON chemotype in both populations (Fig. 2).

**Table 2:** Means and standard errors for FHB severity assessed in the greenhouse at 14 days post inoculation (GH14), 21 days post inoculation (GH21), and area under disease progress curve (GH_AUDPC) in gene classes and check lines for CDC Alsask and CDC Go near-isogenic lines. Means within each column for each population followed by the same letter are not statistically significantly different according to Fisher’s least significant difference (LSD) at *P* = 0.05

Gene/genotype	GH14 (%)			GH21 (%)			GH_AUDPC		
	Mean^a^	SEM^b^	PDR^c^	Mean	SEM	PDR	Mean	SEM	PDR
*CDC Alsask*									
CDC Teal (susceptible check)	72.7 a	2.3	–	98.6 a	1.1	–	977.3 a	26.9	–
CDC Alsask (recurrent parent)	59.1 bc	1.8	–	97.2 a	1.5	–	862.0 b	21.6	–
AC Barrie (moderately resistant/intermediate check)	49.1 d-g	3.3	–	83.3 b	2.0	–	676.3 de	32.1	–
ND2710 (resistant check)	7.4 i	1.6	–	15.4 d	4.1	–	124.2 g	24.1	–
04GC0139 (resistance donor parent)	6.1 i	1.4	89.7	7.6 d	1.3	92.2	87.9 g	14.7	89.8
Null (*n* = 4)	55.3 bcd	1.6	6.4	91.3 ab	1.9	6.1	749.3 c	18.1	13.1
*Fhb1* (*n* = 6)	42.2 gh	2.0	28.6	82.4 b	2.5	15.2	607.2 ef	22.8	29.6
*Fhb2* (*n* = 4)	47.6 c-g	2.0	19.5	88.0 b	2.5	9.5	673.3 d	22.1	21.9
*Fhb5* (*n* = 2)	44.8 e-h	3.0	24.2	80.1 bc	3.9	17.6	613.9 def	33.4	28.8
*Fhb1* + *Fhb2* (n = 2)	48.4 b-h	4.5	18.1	86.6 bc	4.1	10.9	685.5 cde	50.1	20.5
*Fhb1* + *Fhb5* (n = 4)	50.0 b-e	2.4	15.4	82.1 bc	2.0	15.5	666.9 de	22.9	22.6
*Fhb2* + *Fhb5* (n = 6)	47.2 d-f	1.4	20.1	81.1 bc	1.7	16.6	640.9 de	14.1	25.6
*Fhb1* + *Fhb2* + *Fhb5* (n = 4)	37.0 h	3.5	37.4	70.9 c	4.7	27.1	529.8 f	39.2	38.5
*CDC Go*									
CDC Teal (susceptible check)	72.7 a	7.1	–	98.7 a-d	7.2	–	977.6 a	68.9	–
CDC Go (recurrent parent)	78.0 a	7.1	–	100.0 a-d	7.2	–	917.8 ab	68.9	–
AC Barrie (moderately resistant/intermediate check)	49.1 bc	7.1	–	83.3 bcd	7.2	–	676.6 cd	68.9	–
ND2710 (resistant check)	7.4 e	7.1	–	15.5 f	7.1	–	124.5 f	68.9	–
04GC0139 (resistance donor parent)	5.9 e	7.1	92.4	7.9 f	7.1	92.1	87.6 f	68.9	90.5
Null (*n* = 7)	67.9 a	2.9	12.9	99.0 a	2.7	1.0	844.5 ab	27.5	8.0
*Fhb1* (n = 6)	63.4 b	3.1	18.7	95.8 abc	3.0	4.2	805.6 bc	29.5	11.1
*Fhb2* (n = 4)	68.2 a	3.7	12.6	97.5 ab	3.6	2.5	848.6 ab	35.4	7.5
*Fhb5* (n = 2)	53.1 bc	5.1	31.9	83.2 d	5.1	16.8	686.6 d	49.2	25.2
*Fhb1* + *Fhb2* (n = 4)	45.8 c	3.7	41.3	88.6 bcd	3.6	11.4	653.8 d	35.5	28.8
*Fhb1* + *Fhb5* (n = 6)	47.2 c	3.1	39.5	87.0 d	3.0	13.0	657.4 d	29.5	28.4
*Fhb2* + *Fhb5* (n = 4)	52.4 c	3.8	32.8	87.1 cd	3.7	12.9	693.9 d	36.0	24.4
*Fhb1* + *Fhb2* + *Fhb5* (*n* = 5)	29.8 d	3.4	61.8	67.1 e	3.2	32.9	464.8 e	32.0	49.4

^a^Least squares mean; ^b^Standard error of the mean; ^c^Percent disease reduction compared to recurrent parent

In the field evaluation of the CDC Alsask population, only gene combinations of *Fhb2 + Fhb5* and *Fhb1 + Fhb2 + Fhb5* reduced FHB incidence and all three genes or their combinations reduced severity and FHB index compared to the recurrent parent (Table 3). The DON toxin was reduced only by *Fhb1 + Fhb5*, *Fhb2 + Fhb5* and *Fhb1 + Fhb2 + Fhb5*. For CDC Go, only the combination of all three genes reduced incidence and index, whereas all other gene classes were comparable to CDC Go. The DON accumulation was reduced by *Fhb5* and combinations of *Fbh5* with *Fhb1* and *Fhb2*, as did *Fhb1 + Fhb2 + Fhb5*. The combination of these three genes reduced FLD_INC, FLD_SEV, FLD_IND, and FLD_DON by 9, 32, 37, and 49% in the CDC Alsask background, and by 14, 20, 26, 40% in the CDC Go background, respectively.

**Table 3:** Means and standard errors for field incidence (FLD_INC), field severity (FLD_SEV), field FHB index (FLD_IND), and deoxynivalenol (FLD_DON) accumulation in gene classes and check lines for CDC Alsask and CDC Go near-isogenic lines. The data is combined over 6 environments. Means within each column for each population followed by same letter are not statistically significantly different according to Fisher’s least significant difference (LSD) at *P* = 0.05

Gene/genotype	FLD_INC (%)			FLD_SEV (%)			FLD_IND (%)			FLD_DON (ppm)		
	Mean^a^	SEM^b^	PDR^c^	Mean	SEM	PDR	Mean	SEM	PDR	Mean	SEM	PDR
*CDC Alsask*												
CDC Teal (susceptible check)	78.7 a	4.3	–	61.7 a	8.4	–	51.1 a	6.9	–	43.6 a	8.7	–
CDC Alsask (recurrent parent)	76.0 ab	4.4	–	62.7 a	8.4	–	48.8 a	7.0	–	34.8 ab	8.7	–
AC Barrie (moderately resistant/intermediate check)	58.6 d	4.7	–	37.1 f	8.4	–	22.1 d	7.0	–	18.4 cd	6.4	–
ND2710 (resistant check)	50.2 de	4.7	–	18.8 g	7.0	–	10.0 e	4.0	–	9.5 d	4.0	–
04GC0139 (resistance donor parent)	48.4 e	4.3	36.3	25.7 g	7.1	59.0	12.2 e	4.1	75.0	9.5 d	4.0	72.7
Null (n = 4)	74.0 abc	3.7	2.6	49.8 bc	7.9	20.6	37.8 b	6.8	22.5	27.4 bc	8.4	21.3
*Fhb1* (n = 6)	75.6 ab	3.6	0.5	47.0 cd	7.8	25.0	36.5 bc	6.7	25.2	26.2 bc	8.3	24.7
*Fhb2* (n = 4)	74.2 abc	3.7	2.4	51.3 b	7.9	18.2	39.8 b	6.8	18.4	30.4 b	8.4	12.6
*Fhb5* (n = 2)	74.8 abc	3.8	1.6	48.3 bcd	7.9	23.0	36.7 bc	6.8	24.8	23.6 bc	8.4	32.2
*Fhb1* + *Fhb2* (n = 2)	74.6 abc	3.9	1.8	46.1 cde	8.0	26.5	35.2 bc	6.8	27.9	26.4 bc	8.5	24.1
*Fhb1* + *Fhb5* (n = 4)	72.7 abc	3.7	4.3	47.4 bcd	7.9	24.4	36.2 bc	6.8	25.8	19.9 cd	8.1	42.8
*Fhb2* + *Fhb5* (n = 6)	68.3 c	3.6	10.1	44.6 de	7.8	28.9	31.3 c	6.8	35.9	19.9 cd	8.0	42.8
*Fhb1* + *Fhb2* + *Fhb5* (n = 4)	69.1 c	3.6	9.1	42.8 ef	7.9	31.7	30.6 cd	6.7	37.3	17.6 cd	8.0	49.4
*CDC Go*												
CDC Teal (susceptible check)	76.7 a	8.0	–	67.7 a	7.4	–	51.7 a	7.9	–	32.2 a	6.8	–
CDC Go (recurrent parent)	75.6 ab	8.0	–	46.6 b	7.4	–	37.2 b	7.9	–	30.6 a	6.7	–
AC Barrie (moderately resistant/intermediate check)	55.1 d	8.1	–	35.7 b	7.6	–	21.1 d	8.0	–	17.4 de	6.8	–
ND2710 (resistant check)	39.1 e	8.1	–	19.4 c	7.6	–	9.7 e	8.0	–	5.3 f	6.9	–
04GC0139 (resistance donor parent)	37.5 e	8.0	50.4	19.1 c	7.4	59.0	7.8 e	7.9	79.0	8.1 f	6.6	73.5
Null (n = 7)	72.9 abc	7.7	3.6	45.4 b	7.1	2.6	35.9 b	7.7	3.5	29.2 a	6.2	4.6
*Fhb1* (n = 6)	70.8 abc	7.7	6.4	43.6 b	7.1	6.4	33.9 bc	7.7	8.9	25.5 abc	6.3	16.1
*Fhb2* (n = 4)	71.2 abc	7.7	5.8	43.0 b	7.2	7.7	33.4 bc	7.7	10.2	28.7 ab	6.3	6.0
*Fhb5* (n = 2)	67.1 abc	7.8	11.2	43.4 b	7.3	7.3	32.8 bc	7.8	11.8	21.4 cde	6.5	30.1
*Fhb1* + *Fhb2* (n = 4)	70.3 abc	7.7	7.0	40.7 b	7.2	12.7	31.4 bc	7.7	15.6	24.6 a-d	6.3	19.6
*Fhb1* + *Fhb5* (n = 6)	66.5 bc	7.7	12.0	40.8 b	7.1	12.4	30.6 bc	7.7	17.7	20.3 cde	6.3	33.7
*Fhb2* + *Fhb5* (n = 4)	69.6 abc	7.7	7.9	42.3 b	7.2	7.9	32.8 bc	7.7	11.8	22.8 b-e	6.3	25.5
*Fhb1* + *Fhb2* + *Fhb5* (n = 5)	65.0 cd	7.7	14.0	37.4 b	7.2	19.7	27.4 cd	7.7	26.3	18.3 e	6.3	40.2

^a^Least squares mean; ^b^Standard error of the mean; ^c^Percent disease reduction compared to recurrent parent

### Marker main effects and epistatic interactions

Epistatic interaction analyses were carried out between marker pairs for all marker loci and multiple genome-wide interactions were identified that influenced FHB parameters (Table 4). Statistically significant epistatic interactions (*P* < 0.001) among other marker loci and *Fhb1*, *Fhb2*, and *Fhb5* were identified; interactions with *Fhb5* were the most common among the three genes, however, the nature of the epistatic interactions (additive/additive-dominant) could not be defined. In the CDC Alsask population, epistatic interactions explained up to 18.1% of the variation for FLD_INC, 24.4% for FLD_SEV, 25.9% for FLD_IND, and 16.4% for FLD_DON accumulation (Table 4). Similarly, for CDC Go, up to 16.6% of the phenotypic variation was explained for GH_AUDPC, 18.7% for FLD_INC, 10.7% for FLD_SEV, 18.9% for FLD_IND, and 22.2% for FLD_DON accumulation. In addition to *Fhb1*, *Fhb2*, and *Fhb5*, four stable alleles (stable alleles in this paper are those identified in multiple environments) in CDC Alsask NILs and five in CDC Go NILs were identified that conferred resistance against FHB (Tables 5 and 6). Of the resistance improving alleles, one of the four in CDC Alsask and three of the five in CDC Go were contributed by the susceptible recurrent parent. One of the four alleles identified in CDC Alsask on chromosome 6A overlapped with QTL *Qfhb.ndwp-6A* reported by Zhao et al. [29]; they mapped the QTL from ND2710, an advanced breeding line from North Dakota, USA and derivative of Sumai 3. The favourable alleles at 1DS, 6AS, *Qfhb.ndwp-6A*, 7BS loci in CDC Alsask reduced FHB index up to 23.4, 15.7, 17.2, and 17.2% and and DON accumulation by as much as 26.0, 13.7, 37.8, and 14.8%, respectively (Table 5). In CDC Go, the favourable alleles at 1DL, 2AL, 2DL, 6DS, 7AL loci reduced FHB index up to 19.0, 23.2, 16.7, 22.3, and 5.6% and DON accumulation up to 24.5, 19.4, 15.1, 18.8, and 18.8%, respectively (Table 6).

**Table 4:** Significant (*P* = 0.001) epistatic marker-marker interactions and percent phenotypic variation explained (*R*^*2*^) by the interaction in CDC Alsask and CDC Go near-isogenic lines (NILs). Here AA and BB alleles are from recurrent parents CDC Go/CDC Alsask and resistance donor parent 04GC0139, respectively

Trait	Chromosome-Marker/loci and alleles (in parentheses) involved	*R* ^2^
*CDC Alsask*		
INC	6A-Ku_c1976_663 (AA), 5B-wsnp_Ku_c12464_20125626 (AA)	18.1
	6A-Excalibur_c18333_175 (AA), 5D-IACX6288 (AA)	16.6
	6A-Ku_c1976_663 (AA), 5B-Excalibur_c29304_176 (AA), 5B-tplb0027f13_1493 (AA)	10.6
	5B-wsnp_Ku_c12464_20125626 (AA), 5B-BobWhite_c13340_412 (AA)	5.8
SEV	5D-Excalibur_c34793_1260 (AA), 6A-RAC875_c13610_822 (BB), 6A-BS00071571_51(AA)	24.4
	*Fhb1*-3B-CAP7_c1576_371 (AA), 2D-Excalibur_c73791_215 (AA), 2D-IAAV8570 (AA), 2D-RAC875_c319_1776 (AA)	17.4
	3D-Kukri_rep_c96809_457 (AA), 2D-Excalibur_c73791_215 (AA), 2D-IAAV8570 (AA), 2D-RAC875_c319_1776 (AA)	6.8
IND	*Fhb5*-5A-BS00077990_51 (BB), *Fhb5*-5A-Tdurum_contig10128_593 (BB), *Fhb5*-5A-BS00071087_51 (BB), *Fhb5*-5A-BS00045284_51 (BB), 5A-BS00078572_51 (BB), 5A-BS00078573_51 (BB), 5B-tplb0027f13_1493 (AA), 5B-Excalibur_c29304_176 (AA), 5A-wsnp_Ex_c11309_18272248 (BB)	24.8–25.9
	4B-BS00022582_51 (BB), 4B-BS00022582_51 (BB), 1D-RAC875_c10387_685 (AA), 1D-Kukri_c26168_713 (AA), 1D-BobWhite_c1715_887 (AA), 1D-Excalibur_c15692_532 (AA), Un-BS00064204_51 (AA), 1A-Kukri_c29150_143 (AA)	20.7
	5B-Ex_c5594_2630 (AA), *Fhb5*-5A-BS00077990_51(BB), *Fhb5*-5A-Tdurum_contig10128_593(BB), *Fhb5*-5A-BS00071087_51(BB), *Fhb5*-5A-BS00045284_51 (BB), 5A-BS00078573_51 (BB), 5A-Ra_c322_1259 (BB), 5A-BS00078572_51 (BB), 5A-GENE-3218_77 (BB), 5A-wsnp_Ex_c11309_18272248 (BB)	7.8–19.7
	4A-wsnp_Ku_c4342_7887834 (BB), 2B-wsnp_Ex_c17576_26303707 (BB), 7B-CAP11_c8077_69 (AA)	6.8–12.6
	6B-Tdurum_contig81911_179 (BB), 1A-Kukri_c23985_166 (BB), 1A-Excalibur_c75270_566 (BB), 1A-Tdurum_contig43646_147 (BB)	12.4
DON	7A-Excalibur_c52972_213 (AA), *Fhb5*-5A-BS00077990_51 (AA), *Fhb5*-5A-BS00071087_51 (AA), 5A-wsnp_Ex_c11309_18272248 (AA), 6B-Tdurum_contig42203_3670 (AA)	3.3–16.4
	5B-Ex_c5594_2630 (AA), 1B-Tdurum_contig893_53 (BB), 1A-Tdurum_contig5560_193 (BB), 1B-Tdurum_contig42558_134 (BB)	15.5
	5A-BS00078572_51 (AB), 6B-Tdurum_contig81911_179 (AA)	15.2
	*Fhb5*-5A-BS00077990_51 (AA), *Fhb5*-5A-Tdurum_contig10128_593 (AA), *Fhb5*-5A-BS00071087_51 (AA), *Fhb5*-5A-BS00045284_51 (AA), *Fhb1*-3B-BS00063445_51 (AA), *Fhb2*-6B-wsnp_Ex_c5058_8981554 (BB), *Fhb2*-6B-Kukri_c66290_127, 5A-wsnp_Ex_c11309_18272248 (AA), 2B-BobWhite_rep_c49523_266 (BB), 3B-BS00001335_51 (AA), 6D-Excalibur_c17241_388 (BB), 3A-BS00036089_51 (AA), 3D-Kukri_rep_c111139_338 (BB)	5.7–8.4
	7B-BS00063852_51 (AA), 1D-BS00015317_51 (BB), 1D-Excalibur_c15692_53 (BB), 1D-RAC875_c10387_685 (BB)	6.5
	*Fhb5*-5A-BS00077990_51 (AA), *Fhb5*-5A-Tdurum_contig10128_593 (AA), *Fhb5*-5A-BS00071087_51 (AA), *Fhb5*-5A-BS00045284_51 (AA), *Fhb1*-3B-BS00063445_51 (AA), Un-BS00064204_51 (AA), 1A-Kukri_c29150_143 (AA), 1D-Kukri_c26168_713 (AA), 1D-BobWhite_c1715_887 (AA), 1D-Excalibur_c15692_532 (AA)	3.2–6.2
	*Fhb2*-6B-wsnp_Ex_c5058_8981554 (BB), 6D-Excalibur_c17241_388 (BB), 6B-Tdurum_contig42203_3670 (AA)	6.1
*CDC Go*		
GH_AUDPC	*Fhb5*-5A-wsnp_Ra_rep_c69221_66574148 (AA), 2A-Tdurum_contig21786_270 (AA), 2D-IACX8602 (AA), 7D-RAC875_c11969_384 (AA), 4D-wsnp_JD_rep_c51623_35119179 (AA), 7A-RAC875_c22592_2255 (AA), 7D-tplb0041e14_1096 (AA)	8.8–16.6
	*Fhb5*-5A-wsnp_Ra_rep_c69221_66574148 (BB), *Fhb5*-5A-BS00045284_51 (BB), 7D-RAC875_c11969_384 (AA), 4D-wsnp_JD_rep_c51623_35119179 (AA), 7D-tplb0041e14_1096 (AA)	16.6
INC	*Fhb5*-5A-BS00045284_51 (BB), *Fhb5*-5A-barc186 (BB), *Fhb5*-5A-BS00041219_51 (BB), *Fhb5*-5A-barc117 (BB), *Fhb5*-5A-wsnp_Ra_rep_c69221_66574148 (BB), 2A-wsnp_Ex_c36481_44425685 (AA), 2D-Excalibur_c65796_394 (AA)	11.0–18.7
	*Fhb5*-5A-BS00045284_51 (AA), *Fhb5*-5A-barc186 (AA), *Fhb5*-5A-wmc705 (AA), *Fhb5*-5A-barc117 (AA), *Fhb5*-5A-wsnp_Ra_rep_c69221_66574148 (AA), *Fhb1*-3B-RAC875_c4389_1412 (BB), 7A-Excalibur_c61603_1052 (AA)	11.9–16.6
	*Fhb5*-5A-BS00041219_51 (AA), *Fhb5*-5A-barc117 (AA)	12.6
	*Fhb5*-5A-BS00041219_51 (BB), *Fhb5*-5A-wsnp_Ra_rep_c69221_66574148 (BB), 1A-IAAV4238 (AA), 1B-Excalibur_c35289_64 (AA), 1D-Excalibur_c26495_84 (AA), 5B-Kukri_c6176_1400 (AA)	9.2–12.7
	*Fhb2*-6B-Ra_c3381_1027 (AA), 2A-wsnp_Ex_c36481_44425685 (BB), 6D-BS00110365_51 (AA)	9.4
SEV	*Fhb1*-RAC875_c4389_1412 (BB), *Fhb2*-6B-Ra_c3381_1027 (AA), 6D-BS00110365_51 (AA)	9.3–10.1
	*Fhb2*-6B-Ra_c3381_1027 (AA), 3B-wsnp_JD_c222_352320 (BB), 6D-BS00110365_51 (AA)	10.1
	2A-wsnp_Ex_c36481_44425685 (BB), 7B-GENE-4981_53 (BB), *Fhb2*-6B-Ra_c3381_1027 (AA)	10.2
	7A-wsnp_Ex_c39221_46569987 (AA), *Fhb5*-5A-barc186 (AA), *Fhb5*-5A-BS00041219_51 (AA)	8.2–10.7
IND	*Fhb5*-5A-wsnp_Ra_rep_c69221_66574148 (BB), *Fhb5*-5A-BS00045284_51 (BB), *Fhb5*-5A-barc117 (AA), *Fhb5*-5A-wmc705 (AA), *Fhb2*-6B-Ra_c3381_1027 (AA), 2A-wsnp_Ex_c36481_44425685 (BB), 6A-Kukri_c56494_585 (BB), 2A-Tdurum_contig21786_270 (AA), 2D-IACX8602 (AA), 6D-BS00110365_51 (AA)	17.0–18.9
	1A-IAAV4238 (BB), 1B-Excalibur_c35289_64 (BB), 2D-Excalibur_c65796_394 (BB), 5B-BobWhite_c11038_605 (BB)	15.5
	*Fhb1*-RAC875_c4389_1412 (BB), 5B-Kukri_c6176_1400 (BB)	10.1
DON	*Fhb5*-5A-BS00045284_51 (AA), *Fhb5*-5A-barc186 (AA), 7A-Excalibur_c7897_600 (AA)	5.0–22.2
	*Fhb5*-5A-BS00041219_51 (BB), *Fhb5*-5A-wsnp_Ra_rep_c69221_66574148 (BB), *Fhb5*-5A-BS00045284_51 (BB), 7A-Excalibur_c7897_600 (AA), 4D-wsnp_JD_rep_c51623_35119179 (AA), 7D-tplb0041e14_1096 (AA)	10.1–21.3
	*Fhb1*-RAC875_c4389_1412 (AA), *Fhb2*-6B-Ra_c3381_1027 (AA), *Fhb5*-5A-BS00041219_51 (AA), 6D-BS00110365_51 (AA), 3B-wsnp_JD_c222_352320 (AA)	8.1–20.3
	*Fhb5*-5A-wsnp_Ra_rep_c69221_66574148 (AA), 1A-IAAV4238 (BB)	14.8
	*Fhb5*-5A-wsnp_Ra_rep_c69221_66574148 (AA), *Fhb5*-5A-barc186 (AA), *Fhb1*-RAC875_c4389_1412 (AA), 3B-wsnp_JD_c222_352320 (AA)	5.6–9.9

**Table 5:** Single nucleotide polymorphism (SNP) markers (other than *Fhb1*, *Fhb2*, and *Fhb5*) associated (*P* < 0.05) with Fusarium head blight index (FLD_IND), severity (FLD_SEV), incidence (FLD_INC), deoxynivalenol accumulation (FLD_DON), and area under disease progress curve from greenhouse evaluation (GH_AUDPC) in CDC Alsask near-isogenic lines. The numbers not in parentheses represent differences (in units for the traits) in LSmeans and the number in parentheses indicate percent disease reduction relative to susceptible allele

Chr./Locus (allele)^a^	Physical interval (Mb)	2011^b^	2012	2013	2015	2016	2016S	Average	GH_AUDPC
FLD_IND									
1DS (BB)	10.39–26.18	–	3.2 (7.2)	6.9 (23.4)	–	3.6 (23.2)	–	2.0 (5.6)	105.1 (15.9)
6AS (BB)	3.35	5.3 (8.0)	3.8 (11.2)	4.0 (13.6)	7.8 (15.7)	–	–	3.3 (9.1)	–
*Qfhb.ndwp-6A*^b^ (BB)	602.5–611. 8	–	–	3.7 (11.8)		3.0 (17.2)	–	–	–
7BS (AA)	170.52	4.3 (6.4)	3.5 (11.6)	–	9.1 (17.2)	–	3.2 (12.2)	3.5 (9.3)	68.9 (10.0)
FLD_SEV									**–**
1DS (BB)	10.39–26.18	–	2.9 (6.7)	7.9 (17.6)	2.4 (3.7)	5.7 (23.3)	–	3.2 (6.7)	–
6AS (BB)	3.35	–	9.3 (20.1)	–	4.4 (6.8)	–	–	–	–
*Qfhb.ndwp-6A* (BB)	602.5–611. 8	–	–	9.4 (18.3)	–	–	3.3 (12.5)	–	–
7BS (AA)	170.52	–	4.9 (10.5)	–	4.9 (7.3)	–	2.8 (8.4)	2.6 (5.4)	–
FLD_INC									
1DS (BB)	10.39–26.18	–	3.8 (5.0)	3.5 (5.5)	–	–	–	–	–
6AS (BB)	3.35	5.2 (6.1)	–	8.3 (12.6)	7.7 (10.2)	–	–	–	–
*Qfhb.ndwp-6A* (BB)	602.5–611. 8	–	5.9 (7.4)	–	–	–	–	–	–
7BS (AA)	170.52	4.1 (4.7)	–	7.9 (11.6)	8.8 (11.2)	–	–	–	–
FLD_DON									
1DS (BB)	10.39–26.18	12.8 (23.8)	9.8 (24.3)	5.1 (23.6)	2.6 (9.9)	–	1.3 (26.0)	4.5 (18.1)	–
6AS (BB)	3.35	–	5.5 (13.7)	–	3.7 (13.7)	–	–	–	–
*Qfhb.ndwp-6A* (BB)	602.5–611. 8	10.4 (17.3)	11.4 (23.8)	2.3 (10.1)	–	–	1.4 (37.8)		–
7BS (AA)	170.52	–	4.2 (10.1)	3.4 (14.8)	2.8 (10.1)	–	–	–	–

^a^Here AA and BB in parentheses indicates the CDC Alsask (recurrent susceptible parent) or 04GC0139 (resistance donor parent) alleles, respectively, that contribute resistance

^b^Refer to Zhao et al. (2018)

**Table 6:** Single nucleotide polymorphism (SNP) markers (other than *Fhb1*, *Fhb2*, and *Fhb5*) significantly associated (*P* < 0.05) with Fusarium head blight index (FLD_IND), severity (FLD_SEV), incidence (FLD_INC), deoxynivalenol accumulation (FLD_DON), and area under disease progress curve from greenhouse evaluation (GH_AUDPC) in CDC Go near-isogenic lines. The numbers not in parentheses represent differences (in units for the traits) in LSmeans and the number in parentheses indicates percent disease reduction relative to susceptible allele

Chr./Locus (allele)^a^	Position (Mb)	2010^b^	2011	2012	2013	2015	2016	2016S	Average	GH_AUDPC
FLD_IND										
1DL (AA)	492.17–495.11	3.3 (19.0)	–	–	4.1 (6.6)	3.6 (5.7)	–	–	–	80.1 (10.5)
2AL (AA)	717.8–718.73	4.1 (23.2)	6.1 (13.5)	4.9 (16.1)	–	3.4 (5.4)	–	–	3.1 (9.2)	100.4 (13.0)
2DL (AA)	293.13	2.8 (16.7)	2.4 (5.6)	4.5 (15.2)	–	3.9 (6.2)	2.1 (15.2)	–	2.2 (6.6)	95.3 (12.5)
6DS (BB)	72.01–136.12	–	–	6.8 (22.3)	4.6 (7.5)	4.0 (6.3)	–	–	2.6 (7.8)	116.7 (15.3)
7AL (BB)	519.96–619.15	–	–	–	3.4 (5.6)	–	–	–	–	–
FLD_SEV										
1DL (AA)	492.17–495.11	3.8 (13.9)	–	–	–	–	–	–	–	–
2AL (AA)	717.8–718.73	5.4 (19.3)	5.0 (9.3)	3.8 (8.1)	–	–	–	–	2.6 (6.0)	–
2DL (AA)	293.13	4.4 (16.1)	3.5 (6.5)	2.8 (6.2)	–	–	–	–	–	–
6DS (BB)	72.01–136.12	–	–	6.7 (14.1)	4.4 (6.2)	4.3 (6.7)	4.0 (19.5)	–	3.0 (6.9)	–
FLD_INC										
1DL (AA)	492.17–495.11	4.4 (7.2)	–	–	–	–	–	–	–	–
2AL (AA)	717.8–718.73	–	4.2 (5.0)	6.7 (10.4)	–	–	4.8 (6.9)	2.4 (6.7)	3.2 (4.5)	–
2DL (AA)	293.13	–	–	5.1 (8.1)	–	–	5.4 (7.8)	–	–	–
6DS (BB)	72.01–136.12	–	4.6 (7.3)	–	–	–	4.5 (6.6)	–	–	–
7AL (BB)	519.96–619.15	5.0 (8.3)	–	–	–	–	–	–	–	–
FLD_DON										
1DL (AA)	492.17–495.11	4.4 (18.3)	8.5 (14.0)	1.5 (5.3)	–	4.0 (13.8)	–	1.2 (24.5)	3.0 (11.5)	–
2AL (AA)	717.8–718.73	2.6 (11.6)	5.6 (9.5)	4.2 (14.1)	6.3 (19.4)	–	–	–	2.6 (10.1)	–
2DL (AA)	293.13	–	–	4.1 (13.9)	4.7 (15.1)	1.5 (5.5)	–	0.7 (11.4)	–	–
6DS (BB)	72.01–136.12	–	7.3 (12.4)	–	–	–	1.2 (18.8)	0.8 (17.8)	–	–
7AL (BB)	519.96–619.15	2.6 (11.7)	11.2 (18.8)	–	–	2.1 (7.7)	–	–	2.4 (9.6)	–

^a^Here AA and BB in parentheses indicates the CDC Go (recurrent susceptible parent) or 04GC0139 (resistance donor parent) alleles, respectively, that contribute resistance

### Physical mapping and functional annotation

To determine the physical location of all genes/loci associated with FHB resistance, the corresponding SNP marker sequences were used. As expected, *Fhb1* was located on the distal end of the Chromosome 3B between 8 and 21 Mb; *Fhb2* between 159 and 234 Mb; *Fhb5* in region between 46 and 111 Mb (Fig. 1). Other than FHB major genes, all other regions were physically located in a narrow interval with the exception of 6DS and 7AL in the CDC Go population (Table 6). Although expressed genes were retrieved for all FHB resistance governing regions, we have reported only the annotated genes for regions other than *Fhb1*, *Fhb2*, and *Fhb5* (Table 7; Additional file 2). For functional annotation of expressed genes in *Fhb1*, *Fhb2*, and *Fhb5* regions, readers are directed to studies by Rawat et al. [30], Dhokane et al. [31], Samad-Zamini et al. [32], and Schweiger et al. [33, 34]. Of the 33 expressed genes in the *Fhb1* region, reported by Schweiger et al. [34], only six were found in the Chinese Spring database through POTAGE; whereas all six candidates for *Fhb2* reported by Dhokane et al. [31] were retrieved from POTAGE (data not presented). Using POTAGE and POSEQ recombination bin carrying SNP markers associated with the Chromosome regions 1DS, *Qfhb.ndwp-6A*, 1DL, 2AL, 2DL, 6DS, and 7AL, a total of 70, 147, 3, 85, 515, 416, and 161 expressed genes were retrieved (Additional file 2); however, in Table 7 we present only those expressed genes that were directly associated with the 90 K SNP marker sequences. The most important annotated genes, which could be the potential candidates for FHB resistance governing regions were disease resistance proteins, protein kinases (including mitogen-activated protein kinases), disease resistance nucleotide binding sites and leucine rich repeats (NBS-LRR), glutathione syntetase, dihydroflavonol 4-reductase (involved in flavonoid biosynthesis), glycosyl transferases, NAC domain containing protein and F-box domain containing proteins (Table 7; Additional file 2) as these have been reported to play a role in disease resistance (FHB and diseases of other crops) [31, 33–36].

**Table 7:** List of genes (gene ID and name) annotated for single nucleotide polymorphism (SNP) loci conferring resistance to Fusarium head blight (FHB). For each annotated gene, Munich Information Center for Protein Sequences (MIPS) annotation hit ID is provided

Locus associated with FHB resistance	Gene ID	Gene name	MIPS annotation hit ID	Comments^c^
1DS 6AS	Traes_1DS_BDACE1560	Disease resistance protein	sp|Q9T048|DRL27_ARATH	Expressed in all plant parts
	Traes_1DS_F3F17A72C	Protein kinase superfamily protein	AT5G28080.2	High expression in stem and spike (Z32, Z39, Z65)
	Traes_1DS_205D3AC8B	Disease resistance protein CC-NBS-LRR class family	AT5G63020.1	Highly expressed in spike (Z39)
	Traes_1DS_4E3A925B9	Leucine-rich repeat receptor-like protein kinase family protein	AT4G08850.1	Expressed in all plant parts
	Traes_6AS_318DA417A	Protein kinase	AT3G25490.1	Only expressed in leaf (Z23, Z71), stem (Z65), and spike (Z65)
*Qfhb.ndwp-6A* ^a^	Traes_6AL_90B062F76	F-box/RNI-like superfamily protein	AT3G26922.1	Highly expressed in spike (all stages)
	Traes_6AL_8A5E06C77	LRR receptor-like serine/threonine-protein kinase	UniRef90_M8CZR7	Expressed in all plant parts except root
	Traes_6AL_1B43FE620	Lysine-specific histone demethylase 1 homolog 3	sp|Q9CAE3|LDL3_ARATH	Highest expression in spike (Z32 and Z65)
	Traes_6AL_8BA1FF8B2	NAC domain containing protein 2	AT5G04410.1	Expressed in all plant parts
	Traes_6AL_F759812CF	Acyl-CoA-binding domain-containing protein 4	sp|Q9MA55|ACBP4_ARATH	Expressed in all plant parts
7BS	-^b^	–	–	–
1DL	Traes_1DL_63D5C4C8E	Histone deacetylase	AT5G22650.1	Highly expressed in root, stem, and spike (Z65)
2AL	Traes_2AL_57CC2BFDD	dihydroflavonol 4-reductase	AT5G42800.1	Only expressed in grain (Z71)
	Traes_2AL_3341560A9	ATP binding protein	UniRef90_UPI0002BC9F6D	Highly expressed in grain (Z85)
	Traes_2AL_D4EED56CE	histone-lysine N-methyltransferase	AT3G21820.1	Highly expressed in grain (Z85)
	Traes_2AL_B01F4C113	polymerase delta 4	AT1G09815.1	Highly expressed in grain (Z75)
2DL	Traes_2DL_2249C5E82	Regulator of chromosome condensation RCC1 family protein	AT5G63860.1	Highest expression in spike (Z65)
6DS	Traes_6DS_352313CDF	glutathione synthetase 2	AT5G27380.1	Expressed in all parts
	Traes_6DS_A9E719CC8	Pentatricopeptide repeat-containing protein	sp|Q9SVH0|PP329_ARATH	Highly expressed in spike (Z32)
	Traes_6DS_E0FD61378	Unknown	UniRef90_UPI000234F957	Highly expressed in spike (Z32)
	Traes_6DS_C9398DB9C	Synaptotagmin-5	sp|O00445|SYT5_HUMAN	Only expressed in spike (Z65)
	Traes_6DS_78871A7EA	26S protease regulatory subunit 7 homolog A	sp|Q9SSB5|PRS7A_ARATH	Expressed in all parts
	Traes_6DS_762984823	S-adenosylmethionine synthase 4	sp|Q4LB21|METK4_HORVU	Highly expressed in grain (Z85)
7AL	Traes_7AL_13DE4FF55	Lipase 1	sp|P17573|LIP1_GEOCN	Highly expressed in leaf and spike (Z65)
	Traes_7AL_677F233CE	F-box domain containing protein	UniRef90_Q7G5F5	Highly expressed in spike and grain (all stages)
	Traes_7AL_F60FF74CA	Rer1 family protein	AT4G39220.1	Expressed in all plant parts
	Traes_7AL_89E0BA362	Peptidyl-prolyl cis-trans isomerase B	sp|Q9TW32|PPIB_DICDI	Expressed in all plant parts
	Traes_7AL_C501CCF17	Similar to RCD (ribose catalytic domain) one 1	AT2G35510.1	Highly expressed in spike and grain (all stages)
	Traes_7AL_530CAE15B	mitogen-activated protein kinase	AT5G19010.1	Expressed in all plant parts
	Traes_7AL_99483DCCC	Mitochondrial substrate carrier family protein	AT2G46320.1	Highly expressed in grain (Z85)
	Traes_7AL_6599B5B49	Disease resistance protein	sp|Q9T048|DRL27_ARATH	Expressed in all plant parts and spike (Z65)
	Traes_7AL_52779A5E2	nodulin MtN21 /EamA-like transporter family protein	AT3G45870.1	Expressed in all plant parts
	Traes_7AL_D45376F32	myb-like transcription factor family protein	AT3G25790.1	Highly expressed in stem and spike (all stages)
	Traes_7AL_2279551BA	putative type 1 membrane protein	AT3G24160.1	Expressed in all plant parts
	Traes_7AL_8895EDF48	Glycosyltransferase family 61 protein	AT3G18180.1	Expressed in all plant parts
	Traes_7AL_8CDD7A174	UDP-sugar pyrophosphorylase	AT5G52560.1	Expressed in all plant parts
	Traes_7AL_F45599D0D	histone acetyltransferase of the CBP family 12	AT1G16710.1	Expressed in all plant parts
	Traes_7AL_432085C4D1	exocyst subunit exo70 family protein G1	AT4G31540.1	Highest expression in spike (Z65)
	Traes_7AL_8783C1471	Zinc finger protein	sp|Q9C9A9|COL7_ARATH	Highly expressed in spike (Z65) and grain
	Traes_7AL_7B680A58E	Leucine-rich repeat receptor-like protein kinase	AT2G33170.1	Expressed in all parts

^a^Refer to Zhao et al. (2018)

^b^No annotation obtained

^c^Here Z32, Z39, Z65, Z71, Z79, Z85 indicates the cereal growth stages. For more information on cereal growth stages, please refer to Lancashire et al. (1991)

## Discussion

In this study, we successfully introgressed Sumai 3 derived *Fhb1*, *Fhb2*, and *Fhb5* genes in two elite hard red spring wheat cultivars (CDC Go and CDC Alsask) using microsatellite markers. Although studies by Pumphrey et al. [18], McCartney et al. [21], Miedaner et al. [23], Salameh et al. [24], and Xue et al. [25] have also reported successful introgression and evaluation of Sumai 3 derived genes in elite wheat cultivars; our study has several advantages. Firstly, many of these studies did not perform repeated backcrossing and rather derived recombinant inbred lines involving multiple parents, which are expected to carry relatively larger proportion of the resistant donor, whereas we performed repeated backcrossing with implementation of markers at each BC cycle. Secondly, all the studies cited evaluated only *Fhb1* and *Fhb5* and ignored *Fhb2*, another well-characterized gene for FHB Type-II resistance [6, 31]. Lastly, we genotyped our NILs with a large number of SNP markers in addition to microsatellite markers and were able to evaluate polymorphism on all chromosomes and the marker-marker interactions based on phenotypic assessment in 8–9 environments. By repeated backcrossing, we were able to reduce the proportion of donor parent alleles to a large extent, which was even lower than the theoretically expected value of 6.25%. Similar results for introgression of four FHB resistance QTL were reported in Xue et al. [25] although their results could be biased as they used only 150 microsatellite markers. The results from our study and Xue et al. [25] indicated that the MAS is not only helpful in foreground selection of resistance genes or QTL, but to retain a major portion of the recurrent parent’s chromatin. As NILs in both populations, particularly CDC Go, carried < 3% of the donor parent’s genome, we can reliably quantify the allelic effects of *Fhb1*, *Fhb2*, and *Fhb5* in our populations.

Theoretically, the variation in allelic composition of NILs is expected only for the chromosome carrying the gene of interest, but that is practically impossible, especially when microsatellites are used for selection that targets multiple sites in the genome of allopolyploids such as wheat. Therefore, allelic variation on all chromosomes for given SNP markers was expected. The SNP markers from the wheat 90K assay provided very useful information as they represented polymorphisms on all 21 wheat chromosomes and were uniformly distributed over all chromosomes [26]. A number of SNPs on 3BS (carrying *Fhb1*), 5AS (*Fhb2*), and 6BS (*Fhb2*), including those mapped in gene/QTL intervals were located together (physically) on the chromosome arms and inherited together as a haplotype block (Fig. 1), which could be attributed to strong linkage disequilibrium among markers. In particular, *Fhb1* and *Fhb5* were relatively large haplotype blocks with suppressed recombination; *Fhb1* is a diverse haplotype from susceptible spring wheat lines including Chinese Spring [28, 34]. The *Fhb5* gene was fine-mapped to the low recombination peri-centromeric region of chromosome 5A and the SNPs in the gene interval were all mapped to the same region in our populations; polymorphism was absent for most of the chromosome region validating results of successful introgression of *Fhb5*. Low recombination frequencies in *Fhb1* and *Fhb5* regions could be another reason for the relatively large physical segments carrying exactly the same marker haplotypes on 3BS and 5AS.

Unlike most other studies where NIL/entry nested within gene/QTL class had significant variance estimates, our study indicated insignificant variation among NILs within the same QTL class (Table 1). Alternatively, all NILs carrying the same QTL behaved similarly in our populations. These results indicated that there was no or negligible recombination between the markers used for foreground selection and the gene under selection. Loss of target QTL/genes on successful backcrossing is quite possible (because of double crossover events), however, all three genes were recovered in both backgrounds, possibly by using multiple microsatellites flanking the genes at each BC cycle. Also, repeated backcrossing and a very small proportion of the resistant donor could have resulted in less confounding effects from other alleles inherited along with the three major genes under selection. Moderate to high heritability estimates for all FHB parameters suggested that a large proportion of the differences observed among the NILs has a genetic basis. Heritability estimates in GH evaluations were particularly strong, which was not surprising as the environmental variation was minimal in these cases. As expected, the 3-ADON chemotype of *Fg* resulted in higher FHB severity as compared to the 15-ADON chemotype in GH evaluations in both populations because the 3-ADON chemotype is known to be more aggressive and produces more DON than the 15-ADON chemotype [19, 20, 37]. Despite the fact that the 3-ADON chemotype resulted in higher disease severity, the difference between 3-ADON and 15-ADON chemotypes was not significant 21 days post inoculation because resistance to FHB in wheat was not complete and the Sumai 3 genes only slow fungal progression. With time (by 21 days after inoculation), both resistant and susceptible spikes will exhibit FHB symptoms, particularly under conducive conditions coupled with artificial inoculations.

Despite the tendency towards reduced FHB symptoms (incidence, severity, and/or index) and DON accumulation in NILs carrying Sumai 3 derived genes, it was not significant for most of the gene classes in the CDC Go population. This may have been due to the relatively higher level of resistance in recurrent parent CDC Go as compared to CDC Alsask [38]. Some level of resistance in CDC Go compared to CDC Alsask was also evident from the fact that CDC Go has three resistance improving alleles, whereas CDC Alsask has only one (Tables 5 and 6). Similar results on insignificant improvement in FHB resistance in winter wheat cultivar ‘Apache’ (MR) were reported on introgression of *Fhb1* and *Fhb5* [24] and with introgression of *Fhb1* in recipient lines carrying good Type-I resistance [18]. Also, Pumphrey et al. [18] did not detect significant differences for FHB disease severity or the proportion of FDK in half of the families contrasting for *Fhb1*. In practice, it is hard to combine all favourable alleles in one genetic background, particularly when both parents carry favourable alleles; there was no NIL entry in either the CDC Go or the CDC Alsask populations that carried all favourable alleles from each parent. Similar to the results reported by Salameh et al. [24], NILs carrying none of three major FHB genes (classified as ‘null’) in our study tended to improve resistance compared to the recurrent parents and the differences were actually significant in the CDC Alsask NILs. The improved resistance of such NILs could be attributed to some other minor favourable loci derived from either parent (Tables 5 and 6). Our study and all studies cited in our paper, report that even after pyramiding *Fhb1*, *Fhb2*, and *Fhb5* in the same background, the improvement did not lead to development of any NIL or RIL as resistant as the donor parent or the resistant check. This indicates that Sumai 3 and its immediate derivatives include multiple other loci conferring FHB resistance. In our study, we identified 2–3 additional loci, derived from the resistant donor parent, but none of the loci were overlapping in both populations, which in part could explain the additional resistance in the NILs. In addition to the Sumai 3 derived chromosome regions/loci identified in our study, Anderon et al. [39] reported QTL (in addition to *Fhb1*, *Fhb2*, *Fhb5*) on 3AL and 6AS, and Zhou et al. [40] on 2B and 7A.

The genes expressed in the chromosome regions associated with resistance include a wide variety of proteins including disease resistance proteins, protein kinases and nucleotide-binding and leucine rich repeat type proteins, which are most commonly associated with resistance to plant pathogens (Table 7; Additional file 2; [35]). The prediction of disease resistance proteins and kinases (highly expressed in spikes) in resistance conferring regions further validated our results and indicated their potential involvement in FHB suppression. Although genes listed in Table 7 are mostly expressed in spikes and/or grain, and are directly associated with marker sequences, these should be considered in future studies with caution because there were many other genes predicted in the regions listed in Additional file 2. The absence of genes that were predicted in the *Fhb1* region were also absent from our POTAGE analyses, which could be attributed to the fact that this region was very diverse in susceptible lines in terms of gene content and size [34].

The corn-spwan and/or spray inoculation method in field FHB nurseries evaluate both Type-I (incidence) and Type-II (severity) resistance, collectively termed as field resistance [3]. Single-floret point inoculation in controlled conditions such as greenhouse or growth cabinets evaluates only Type-II resistance. An important observation from our results was that Sumai 3 genes did not show additive responses for field resistance, particularly in the CDC Go population (Table 3). The expression of *Fhb5*, which is considered to confer mainly Type-I resistance, was as strong as *Fhb1* (Type-II resistance) in both populations, indicating that *Fhb5* may also confers some level of Type-II resistance. The non-additive response of Sumai 3 derived genes or non-significant reduction even upon introgression of major genes such as *Fhb1* or *Fhb5* suggests epistatic or gene-gene interactions, which are often speculated, but overlooked in such studies. With the given marker density and good sample size in both populations, we were able to underpin the markers/genes involved in significant epistatic interactions in both populations that explained > 20% of the phenotypic variation of all FHB parameters. Epistatic marker-marker interactions were previously reported for some other diseases of wheat particularly for stem rust Ug99 resistance [41, 42]; however, it is worth mentioning that interactions reported by Yu et al. [41] explained less than 9% of the phenotypic variation which could be attributed to the nature of resistance in rusts (vertical/qualitative) vs FHB (horizontal/quantitative). Additionally, the role of environment in epistatic interactions and complex traits such as FHB was also significant, which is why epistatic interactions in our study accounted for a relatively large part of the total phenotypic variation [3, 16]. Frequent involvement of Sumai 3 derived genes, particularly *Fhb5*, in epistatic interactions also suggests their critical role in FHB resistance. Although the nature of epistasis could not be determined in our study, the significant involvement of *Fhb1*, *Fhb2*, and *Fhb5* in interactions along with other loci (from both the recurrent and the donor parent) could explain the non-additive phenotypic expression in our populations and possibly other studies.

## Conclusions

The present study has elucidated the effects of Sumai 3 introgressions on FHB disease resistance and resistance to DON accumulation. As next goal, in another study, we also utilized the set of NILs to evaluate the effect (linkage-drag) of introgressed major genes as well as minor loci on agronomic and end-use quality traits [43]. Before breeders can utilize any identified/mapped QTL or gene in their breeding program, validation using MAS is usually warranted because the effect is not always similar in all genetic backgrounds. NILs with improved resistance and phenological similarity to more advanced elite lines can easily be used for MAS in wheat breeding programs. However, the allelic effect on FHB resistance could differ depending on genetic background and complex epistatic interactions, thus affecting expression and penetrance of the genes in the recipient lines. Although our study suggested that improved resistance in lines carrying so-called ‘native’ resistance may not be as much as in S or MS lines, rare transgressive segregants can also be obtained from such cultivars/lines, which in turn again depends on their genetic background. In fact, Sumai 3 itself was a transgressive segregant from its parents [4]. The importance of ‘native’ resistance in local elite cultivars should not be ignored while breeding for FHB resistance in wheat.

## Methods

### NIL development using marker-assisted background selection

F_4_ populations were developed from two backcross populations CDC Go*4/04GC0139 and CDC Alsask*4/04GC0139. Line 04GC0139 (*Triticum aestivum* L.) was derived from Sumai 3 and has a high level of resistance to FHB, kindly provided by Dr. Julian Thomas (retired) of the Cereal Research Centre, Agriculture and Agri-Food Canada, Winnipeg, Manitoba. Line 04GC0139 has ND2710 and BW278 (pedigree: AC Domain*2/Sumai 3) in its pedigree which are both derivatives of Suami3. The line 04GC0139 carries three well-characterized genes for resistance to FHB on chromosomes 3BS (*Fhb1)*, 6BS (*Fhb2)* and 5AS (*Fhb5)* (G.S. Brar, unpublished data). The hard red spring wheat cultivar CDC Go (pedigree: Grandin/SD3055) is moderately susceptible (MS) to FHB, and CDC Alsask (pedigree: AC Elsa/AC Cora) is susceptible (S) (Anonymous 2015). The NILs were developed by backcrossing F_1_ plants to the recurrent parents (CDC Go or CDC Alsask) and the F_1_ at each BC cycle was screened with microsatellite markers flanking *Fhb1*, *Fhb2* and *Fhb5.* Approximately 2100 BC_2_F_1_ and 1300 BC_3_F_1_ hybrid seeds were generated by hand crossing and 90% of the seeds were germinated and haplotyped. During the selfing process, 123 BC_3_F_1_-derived families were advanced and approximately 7000 F_2_ seedlings were grown, which were used to generate seed for F_3_ plots grown in the field in 2009. Two hundred spikes were harvested from each plot and haplotyped in the F_4_ generation. A total of 70 lines (38 from CDC Go and 32 from CDC Alsask) representing all eight possible combinations of FHB genes were recovered.

### Microsatellite and SNP genotyping

Genomic DNA was extracted from grain and/or leaf tissue with the DNeasy 96 Plant Kit (Qiagen, Mississauga, ON). Quantification of DNA was done by fluorometry using Hoechst 33258 stain. During population development, a total of seven simple sequence repeats markers associated with *Fhb1*, *Fhb2*, or *Fhb5* were screened on genomic DNA. Markers *umn10* for *Fhb1* on chromosome *3BS* [44], *gwm133* and *gwm644* for *Fhb2* on chromosome 6BS [7], and *gwm304*, *barc117*, *wmc705* and *gwm293* for *Fhb5* on chromosome 5AS were used [10]. Each SSR primer pair was modified by addition of the M13 sequence to the 5′ end of the forward primer during synthesis. Fluorescent dye (either HEX, FAM or NED) was used to label the universal M13 primer. The PCR reactions consisted of 1.5 μl 10× PCR buffer, 1.5 (or 0) mM of MgCl_2_, 0.2 mM of each dNTP, 0.04 μM of M13 sequence-modified forward primer, 0.16 μM of reverse primer, 0.152 μM of universal dye-labelled M13 primer, 1 U of *Taq* DNA polymerase, and 50 ng of genomic DNA. The total PCR volume was 15 μL. Temperature cycling included 94 °C for 30 s, 56 °C (or 62 °C) for 50 s, 72 °C for 55 s, 94 °C for 30 s, 54 °C (or 60 °C) for 50 s, 72 °C for 55 s, 94 °C for 30 s, 52 °C (or 58 °C) for 50 s, 72 °C for 55 s, 94 °C for 30 s, 50 °C (or 56 °C) for 50 s, 72 °C for 55 s, then 25 cycles of 94 °C for 30 s, 51 °C for 50 s, 72 °C for 55 s, then 1 cycle of 72 °C for 10 min. Primers were first assessed for polymorphism on 2% (*w/v*) agarose gel stained with 0.5 μg/ml ethidium bromide, then further tested for polymorphism by capillary electrophoresis (CE) using an AB13100 Genetic Analyzer (Applied Biosystems). For CE, 1 μL of diluted PCR product (diluted 1/5, 1/10 or 1/20 in deionized water depending on band intensity visualized on agarose gel) was combined with 9.0 μL HiDi formamide (ABI, Foster City, CA, USA) and 0.09 μL of 500 ROX size standard. Samples were run on a 36 cm capillary array, processed with Applied Biosystems Data Collection Software version 2.0, and genotyped using GeneMapper version 3.0. The presence of *Fhb1* in NILs was also confirmed with the KASP assay [44, 45].

The NILs were genotyped with seven microsatellite markers while in the developmental phase in 2008–2011. To confirm the genotype of the NILs, some additional microsatellite markers (from fine-mapping studies reporting a narrow QTL interval) were used in 2017–2018 i.e. *gwm493*, *gwm533*, and functional marker for pore-forming toxin (PFT) protein for *Fhb1* [6, 30], *Fhb2-CAPS3* for *Fhb2* [29], *barc180* and *barc186* for *Fhb5* [28]. Additionally, the NILs were genotyped along with the parents using the wheat 90,000 iSelect assay comprised of 81,587 SNPs [26] to better understand the genomic composition and haplotype structure of the NILs. The SNP alleles were called using GenomeStudio (Illumina Inc., San Diego, CA, USA) and filtered based on polymorphisms between parents.

### Greenhouse FHB evaluations

The 38 CDC Go and 32 CDC Alsask NILs, the parents and a number of check cultivars [CDC Teal as S check, AC Barrie as intermediate (I)/moderately resistant (MR) check, and ND2710 as resistant (R) check] were assessed for FHB symptoms in 2010 in a greenhouse (GH) equipped with incandescent lamps, 16 h photoperiod and 22/16 °C day/night temperatures. Two isolates of *F. graminearum*, M09–07-1, a 3-ADON chemotype (NRRL 52068) and M1–07-2, a 15-ADON chemotype (NRRL 47847), were used for inoculations [19]. At 50% anthesis, a main stem spike (two florets leaving lower two-third of the spike) on each plant was inoculated with a 10 μl macroconidial spore suspension (50,000 spores/ml) containing 0.02% Tween 20. The inoculations were performed as described in [6]. Single floret inoculation was performed to evaluate Type-II resistance. A total of three plants per replication were inoculated and there were three replications in total. The FHB severity was rated as the percentage of infected spikelets per spike at 7 (GH7), 14 (GH14) and 21 days (GH21) post inoculation. Area under disease progress curve (GH_AUDPC), used as a measure of FHB severity over time, was calculated according to [46].

### Field FHB evaluations

The same NILs, parents and check cultivars evaluated in the greenhouse were also assessed in the field nursery at Carman, MB from 2010 to 2013 and 2016, Saskatoon, SK in 2016, and at Morden, MB in 2015. In 2010, the CDC Alsask population was not evaluated in the field because the seed under multiplication in the greenhouse was not ready for field planting. The field trial was set up as a randomized complete block design with two replicates in Carman and four replicates in Morden and Saskatoon. Plots at Morden and Carman consisted of single 1.5 m and 1 m rows, respectively, and in the Saskatoon nursery in hills. Sowing density was approximately 80 seeds per row and 30 seeds per hill. At Carman, every plot in the nursery was artificially inoculated with a suspension of *F. graminearum* macro-conidia prepared with the isolates M9–07-1 (3-ADON), M7–07-1 (3-ADON), M1–07-1 (15-ADON) and M3–07-2 (15-ADON). The isolates used were originally provided by Dr. Jeannie Gilbert at the CRC-AAFC. Isolates were cultured in Spezieller Nährstoffarmer Agar (SNA) for seven days and then incubated in Carboxymethyl Cellulose (CMC) media for another seven to ten days. The number of spores was counted to calculate their concentration. Prior to field application, the suspension of the four isolates was mixed in equal proportions (based on macro-conidia concentration) to provide a total concentration of 50,000 macro-conidia spores/ml. The field application was achieved using a CO_2_ backpack sprayer and directed to the wheat spikes at flowering (anthesis) stage. A second application was performed to the same rows three days later. After each inoculation, plots were mist irrigated overnight. Visual assessments of disease incidence (% of infected spikes in the plot) and severity (% of spikelets infected on the infected spikes) were made on each plot 18–21 days after the first inoculation. Fusarium head blight index for each plot was calculated as follows: (disease incidence x disease severity)/100. At Morden, MB and Saskatoon, SK, irrigated nurseries were inoculated with air-dried corn spawn (colonized by *F. graminearum*) at 50% anthesis. Each plot was assessed using an FHB index (%incidence x %severity/ 100) (FLD_IND) based on disease incidence (%) (FLD_INC) and severity (%) (FLD_SEV) at 21 to 23 dpi. Cultivars CDC Teal (S), AC Barrie (moderately resistant, MR), and ND2710 (resistant, R) were included as checks in Morden and Saskatoon. Cultivars AC Vista, and CDC Teal were used as S check, AC Cora as I check, 5602HR as MR, and FHB37 as R check in Carman nursery. Up to 50 spikes of each NIL were harvested by hand and retained for DON quantification (FLD_DON).

### DON quantification

The spikes of each NIL were harvested from two replicates at the fully ripe stage (BBCH 92; [47]) and dried to minimal water content. Approximately 50–100 g samples of each NIL were ground to a fine powder with a laboratory mill and stored at − 20 °C until further processing. Analysis of DON was carried out using ELISA based assays [48] and a Neogen commercial kit. Measurements were performed in two technical replications of each biological replication. Detailed information on the Neogen ELISA assay are available in Additional file 1 (Protocol #1).

### Physical mapping and functional annotation

All SNP markers from the wheat 90 K assay were physically positioned on the Chinese Spring wheat reference genome sequence. The SNP-bearing sequences were probed to the entire bread wheat NRGene genome assembly RefSeq ver. 1.0 (International Wheat Genome Sequencing Consortium, https://wheat-urgi.versailles.inra.fr/Seq-Repository/Assemblies) using an *in-house* BLAST portal. The best hits, based on sequence similarity and cumulative alignment length percentage of matches, were considered. For annotation, the wheat genome scaffolds carrying the marker were retrieved from the BLAST searches and used to find genes expressed on the scaffolds using POTAGE (PopSeq Ordered *Triticum aestivum* Gene Expression) [49]. POTAGE integrates map location with gene expression, infers functional annotation and visualizes these data through a web browser interface. The map location (implemented in POTAGE) were based on the wheat POPSEQ map of the 90 double haploid individuals of the synthetic W7984 X Opata M85 population, where SNP markers are anchored to contigs in linear order [50].

### SNP data and marker-marker epistatic interaction analyses

For haplotype analyses and to assign each NIL entry to a QTL class, SNP markers tightly linked to SSR markers or mapped to *Fhb1*, *Fhb2*, and *Fhb5* regions were considered [28,29; Ron Knox, unpublished data]. The SNP markers flanking the *Fhb1* region of Carberry were provided by Dr. Ron Knox (AAFC, Swift Current) and were also mapped in ND2710 by Zhao et al. [29]. The introgressed haplotypes from the resistant donor parent were visualized using Graphical Genotypes software ver. 2.0 [51]. To analyze the phenotypic data as influenced by all polymorphic markers, genotypic and phenotypic data were used to test for epistatic interactions. Epistatic interactions between markers with significant main effects (*Fhb1*, *Fhb2*, and *Fhb5*) were tested as well as all other markers regardless of significance. A linear regression model was used to calculate *P*-values for pairwise as well as multiple marker-marker interactions using an *in-house* designed script in the R environment [52]. A false discovery rate of 0.05 was used as a threshold for significant interactions. Epistatic interactions were analyzed according to [53] and modeled as follows:

$$ y=1\mu +{Z}_l{\gamma}_l+\left({Z}_l\times {Z}_{l^{\prime }}\right){\gamma}_{ll^{\prime }}+e $$

Where: *y* is the n × 1 vector for phenotypic observation, *μ* is the population mean, *Z*_1_ is a vector (*Z*_1*l*_ … *Z*_*nl*_)^*T*^, for the genotype indicators of locus *l*, *Z*_*il*_ takes one of two values (− 1, + 1) depending on which parental allele was passed on to line *i*, for locus *l*, *γ*_*l*_ is the additive (main) effect of locus *l*, ${\gamma}_{ll^{\prime }}$ is the epistatic effect between loci *l* and *l*^’^, and *e* is the residual error vector.

From each chromosome, one marker from each group of redundant/co-segregating markers was chosen for the epistatic interaction analyses. Any marker-marker interaction for a given phenotype was declared significant at *P* = 0.001.

### Statistical and phenotypic data analyses

The phenotypic data collected from field and greenhouse evaluations was subjected to correlation and analysis of variance (ANOVA). Before conducting ANOVA, assumptions of independence, normal distribution and homogeneity of residuals for all class variables were verified using Shapiro-Wilk and Levene’s tests implemented in procedure UNIVARIATE in SAS (Statistical Analytical Software) ver. 9.4 (SAS Institute, Inc., Cary, NC). Heterogeneous variances, if any, were modeled using the ‘repeated/group = effect’ statement in procedure MIXED [54]. Variance component estimates and corresponding *F*-values were calculated using the procedure MIXED in SAS ver. 9.4 with the ‘ddfm = kenwardroger’ option to approximate degrees of freedom. Mean separation was conducted using the least significant difference (LSD) test (Fisher’s least significant difference). All tests used a nominal alpha level of 0.05. Broad-sense heritability (*H*^*2*^) was calculated as described in [55]. Pearson’s correlation coefficients among various parameters were calculated using procedure CORR in SAS. Associations among environments, genotypes, and the genotype by environment interaction were analyzed and visualized using biplot analyses [56] in the R environment using the GGEBiplotGUI package [57]. For biplot analyses, the following settings were used: singular value portioning, environment-metric preserving; and genotype by environment scaling, according to the standard deviation; centered by environment (G + G*E).

## Additional files

Additional file 1: **Protocol #1.**Procedure for Neogen enzyme linked immune-sorbent aasay (ELISA) for deoxynivalenol (DON) quantification in Fusarium head blight infected grains. **Table S1.** Proportions of the recurrent parent (RP) and donor parent (DP) genomes in the near-isogenic lines for CDC Go and CDC Alsask streams based on 81,587 SNP markers from 90 K iSelect assay. Here: A, B, H, U represent recurrent parent, donor parent, heterozygous, and unknown alleles, respectively. **Figure S1.** Polymorphism in CDC Go and CDC Alsask near-isogenic lines (NILs). **Figure S2.** Polymorphism in CDC Alsask near-isogenic lines on Chromosomes other than 3B, 5A, 6B. **Figure S3.** Polymorphism in CDC Go near-isogenic lines on Chromosomes other than 3B, 5A, 6B. **Figure S4.** GGE Biplots for CDC Go and CDC Alsask near-isogenic lines (NILs). (DOCX 1474 kb)

Additional file 2: List of annotated genes in the intervals of FHB resistance loci. (XLSX 429 kb)

## Abbreviations

AUDPC: Area under disease progress curve
CDC: Crop Development Centre
DON: Deoxynivalenol
ELISA: Enzyme linked immunosorbant assay
*Fg*: *Fusarium graminearum*
FHB: Fusarium head blight
GH: Greenhouse
INC: Incidence
IND: Index
MAS: Marker-assisted selection
MR: Moderately resistant
MS: Moderately susceptible
NIL: Near-isogenic line
POTAGE: PopSeq Ordered *Triticum aestivum* Gene Expression
QTL: Quantitative trait loci
S: Susceptible
SEV: Severity
SNP: Single nucleotide polymorphism
SSR: Simple sequence repeat
