# Supplementary material for: Genetic factors affecting Fusarium head blight resistance improvement from introgression of exotic Sumai 3 alleles (including Fhb1, Fhb2, and Fhb5) in hard red spring wheat
Source: BMC Plant Biol. 2019 May 3;19:179. doi: 10.1186/s12870-019-1782-2 (PMC6499950; doi:10.1186/s12870-019-1782-2)
Supplement: Supplementary file 1 — Protocol #1.Procedure for Neogen enzyme linked immune-sorbent aasay (ELISA) for deoxynivalenol (DON) quantification in Fusarium head blight infected grains. Table S1. Proportions of the recurrent parent (RP) and donor parent (DP) genomes in the near-isogenic lines for CDC Go and CDC Alsask streams based on 81,587 SNP markers from 90 K iSelect assay. Here: A, B, H, U represent recurrent parent, donor parent, heterozygous, and unknown alleles, respectively. Figure S1. Polymorphism in CDC Go and CDC Alsask near-isogenic lines (NILs). Figure S2. Polymorphism in CDC Alsask near-isogenic lines on Chromosomes other than 3B, 5A, 6B. Figure S3. Polymorphism in CDC Go near-isogenic lines on Chromosomes other than 3B, 5A, 6B. Figure S4. GGE Biplots for CDC Go and CDC Alsask near-isogenic lines (NILs). (DOCX 1474 kb) [file 12870_2019_1782_MOESM1_ESM.docx]

**ADDITIONAL FILE 1**

**Factors affecting Fusarium head blight resistance improvement from introgression of exotic Sumai 3 alleles in hard red spring wheat**

**Gurcharn S. Brar^1^, Anita L. Brûlé-Babel^2^, Yuefeng Ruan^1,3^, Maria Antonia Henriquez^4^, Curtis Jerry Pozniak^1^, Hadley Randal Kutcher^1^, Pierre Jan Hucl^1^**

^1^Crop Development Centre/Department of Plant Science, University of Saskatchewan, 51 Campus Dr., Saskatoon, SK S7N 5A8 Canada

^2^Department of Plant Science, University of Manitoba, 66 Dafoe Road, Winnipeg, MB R3T 2N2 Canada

^3^Present address: Agriculture and Agri-Food Canada, Swift Current Research and Development Centre, 1 Airport Road, Swift Current, SK S9H 3X2 Canada

^4^Agriculture and Agri-Food Canada, Morden Research and Development Centre, 101 Route 100, Morden, MB R6M 1Y5 Canada

***To whom correspondence should be addressed:** E-mail: [pierre.hucl@usask.ca](mailto:pierre.hucl@usask.ca) (Phone: +1-306-966-8667); E-mail: [gurcharn.brar@usask.ca](mailto:gurcharn.brar@usask.ca) (Phone: +1-306-203-1496)

**Protocol #1**

Procedure for Neogen enzyme linked immune-sorbent aasay (ELISA) for deoxynivalenol (DON) quantification in Fusarium head blight infected grains.

**Extraction procedure**

Grind the sample to the consistency of finely ground coffee, or so that at least 75% can pass through a 20-mesh sieve.

1. Weigh out 10 g of the homogenous ground sample into the supplied extraction cup or other suitable receptacle.

2. Add 100 mL of deionized or distilled water to the sample.

3. Shake vigorously for 3 minutes.

4. Filter at least 5 mL of extract through a Neogen filter syringe or a filter funnel and filter paper.

5. Collect the filtered extract using the supplied sample collection tubes. Sample is now ready for testing.

**Test procedure**

1. Warm all reagents to room temperature 18-30 °C (64-86 °F) prior to use.

2. Remove one red-marked mixing well for each sample to be tested, plus five red-marked wells to be used for controls. Place these wells in a microwell holder.

3. Remove an equal number of antibody-coated wells. Immediately return antibody wells that will not be used to the foil pack with desiccant. Reseal the foil pack to protect the antibody. Mark one end of the strip so that the wells can be identified after washing.

4. Mix each reagent by swirling the reagent bottle prior to use.

5. Place 100 μL of conjugate from the blue-labeled bottle in each mixing well.

6. Place 100 μL of each control and sample extraction to the mixing wells as shown below. Use a new pipette tip for each transfer. *(Up to 24 wells may be run at once).*

*Strip 1:* 0 0.5 1 2 6 S1 S2 S3 S4 S5 S6 S7

*Strip 2:* S8 S9 S10 S11 S12 S13 S14 S15 S16 S17 S18 S19

7. Using a 12-channel pipettor, mix the wells by pipetting the liquid up and down in the tips 3-4 times. Transfer 100 μL to the antibody wells and mix by sliding the microwell holder back and forth on a flat surface for 30 seconds. Incubate 2 minutes at room temp. Discard red-marked mixing wells.

8. Dump out the contents of the antibody wells. With a wash bottle or running stream of water, fill each antibody well with deionized or distilled water and then dump the water out. Repeat this step 5 times, then turn wells upside down and tap out on a paper towel until the remaining water has been removed.

9. Pour only the needed volume of substrate from the green-labeled bottle into a clean reagent boat. Using new tips, pipette 100 μL of substrate into the wells and mix 30 seconds. Incubate for 3 minutes at room temp. Discard remaining substrate and rinse the reagent boat with water.

10. Pour the needed volume of red stop solution from the red-labeled bottle into a clean reagent boat. Pipette 100 μL red stop to each well and mix thoroughly by sliding back and forth to eliminate the layering effect. Discard tips.

11. Wipe bottom of microwells with a dry cloth or towel and read within 20 minutes, in a microwell reader using a 650 nm filter.

**Table S1.** Proportions of the recurrent parent (RP) and donor parent (DP) genomes in the near-isogenic lines for CDC Go and CDC Alsask streams based on 81,587 SNP markers from 90K iSelect assay. Here: A, B, H, U represent recurrent parent, donor parent, heterozygous, and unknown alleles, respectively.

|  | | **Total map (14.5 Gb)** | | | | | | **3B (829.5 Mb; carrying *Fhb1*)** | | | | | **5A (709.8 Mb; carrying *Fhb5*)** | | | | **6B (721.0 Mb; carrying *Fhb2*)** | | | |
| --- | --- | --- | --- | --- | --- | --- | --- | --- | --- | --- | --- | --- | --- | --- | --- | --- | --- | --- | --- | --- |
| **Line** | | **A (%)** | | **B (%)** | | **H (%)** | **U (%)** | **A (%)** | **B (%)** | | **H (%)** | **U (%)** | **A (%)** | **B (%)** | **H (%)** | **U (%)** | **A (%)** | **B (%)** | **H (%)** | **U (%)** |
| CDC Go (RP) | | 100.0 | | 0 | | 0 | 0 | 100.0 | 0 | | 0 | 0 | 100.0 | 0 | 0 | 0 | 100.0 | 0 | 0 | 0 |
| 04GC0139 (DP) | | 89.4 | | 10.65 | | 0.00 | 0.00 | 78.1 | 21.95 | | 0.00 | 0.00 | 83.7 | 16.32 | 0.00 | 0.00 | 78.8 | 21.20 | 0.00 | 0.00 |
| Go1 | | 99.2 | | 0.66 | | 0.00 | 0.10 | 98.7 | 1.26 | | 0.00 | 0.08 | 97.6 | 2.34 | 0.00 | 0.06 | 99.8 | 0.11 | 0.00 | 0.08 |
| Go2 | | 99.3 | | 0.68 | | 0.00 | 0.03 | 99.1 | 0.86 | | 0.00 | 0.05 | 94.0 | 5.93 | 0.00 | 0.06 | 99.8 | 0.08 | 0.00 | 0.11 |
| Go3 | | 99.6 | | 0.42 | | 0.00 | 0.02 | 97.8 | 2.17 | | 0.00 | 0.00 | 99.7 | 0.28 | 0.00 | 0.03 | 99.9 | 0.03 | 0.00 | 0.03 |
| Go4 | | 99.1 | | 0.87 | | 0.00 | 0.02 | 99.1 | 0.86 | | 0.00 | 0.05 | 93.3 | 6.68 | 0.00 | 0.03 | 99.7 | 0.19 | 0.00 | 0.08 |
| Go5 | | 99.4 | | 0.59 | | 0.00 | 0.06 | 99.1 | 0.88 | | 0.00 | 0.00 | 99.3 | 0.37 | 0.06 | 0.22 | 99.9 | 0.05 | 0.00 | 0.05 |
| Go6 | | 99.7 | | 0.26 | | 0.03 | 0.06 | 99.7 | 0.19 | | 0.05 | 0.05 | 98.8 | 0.75 | 0.12 | 0.31 | 98.5 | 1.50 | 0.03 | 0.00 |
| Go7 | | 99.3 | | 0.66 | | 0.00 | 0.03 | 99.1 | 0.86 | | 0.00 | 0.08 | 93.2 | 6.68 | 0.00 | 0.09 | 98.3 | 1.61 | 0.03 | 0.05 |
| Go8 | | 99.4 | | 0.61 | | 0.01 | 0.02 | 99.7 | 0.19 | | 0.05 | 0.05 | 93.2 | 6.77 | 0.00 | 0.06 | 98.3 | 1.58 | 0.03 | 0.05 |
| Go9 | | 99.4 | | 0.59 | | 0.01 | 0.03 | 99.7 | 0.19 | | 0.05 | 0.03 | 93.3 | 6.68 | 0.00 | 0.06 | 98.3 | 1.58 | 0.03 | 0.05 |
| Go10 | | 99.7 | | 0.29 | | 0.01 | 0.04 | 98.6 | 1.28 | | 0.05 | 0.03 | 99.6 | 0.34 | 0.00 | 0.03 | 98.4 | 1.53 | 0.03 | 0.08 |
| Go11 | | 99.3 | | 0.65 | | 0.00 | 0.02 | 98.0 | 1.95 | | 0.00 | 0.03 | 93.3 | 6.71 | 0.00 | 0.03 | 99.8 | 0.08 | 0.00 | 0.14 |
| Go12 | | 99.4 | | 0.57 | | 0.00 | 0.08 | 98.8 | 1.12 | | 0.00 | 0.08 | 94.5 | 5.21 | 0.00 | 0.25 | 99.6 | 0.11 | 0.00 | 0.25 |
| Go14 | | 99.5 | | 0.46 | | 0.00 | 0.02 | 100.0 | 0.00 | | 0.00 | 0.03 | 97.3 | 2.62 | 0.00 | 0.03 | 99.9 | 0.03 | 0.00 | 0.08 |
| Go15 | | 99.8 | | 0.18 | | 0.00 | 0.04 | 100.0 | 0.03 | | 0.00 | 0.00 | 99.6 | 0.34 | 0.00 | 0.03 | 99.8 | 0.11 | 0.00 | 0.11 |
| Go16 | | 99.5 | | 0.44 | | 0.00 | 0.09 | 98.7 | 1.28 | | 0.00 | 0.05 | 99.4 | 0.53 | 0.00 | 0.06 | 99.9 | 0.05 | 0.00 | 0.05 |
| Go17 | | 99.6 | | 0.32 | | 0.02 | 0.08 | 99.8 | 0.13 | | 0.00 | 0.03 | 99.3 | 0.53 | 0.00 | 0.12 | 98.4 | 1.50 | 0.03 | 0.08 |
| Go19 | | 99.0 | | 0.93 | | 0.01 | 0.04 | 97.8 | 2.14 | | 0.05 | 0.03 | 93.2 | 6.74 | 0.00 | 0.06 | 99.7 | 0.14 | 0.00 | 0.16 |
| Go20 | | 99.6 | | 0.41 | | 0.00 | 0.03 | 100.0 | 0.00 | | 0.00 | 0.03 | 98.7 | 1.25 | 0.00 | 0.06 | 98.4 | 1.50 | 0.03 | 0.08 |
| Go21 | | 99.2 | | 0.73 | | 0.00 | 0.03 | 100.0 | 0.03 | | 0.00 | 0.00 | 93.1 | 6.86 | 0.00 | 0.03 | 99.8 | 0.11 | 0.00 | 0.08 |
| Go22 | | 99.6 | | 0.34 | | 0.01 | 0.10 | 98.6 | 1.31 | | 0.05 | 0.05 | 99.7 | 0.31 | 0.00 | 0.03 | 99.9 | 0.03 | 0.00 | 0.08 |
| Go23 | | 99.3 | | 0.68 | | 0.01 | 0.04 | 97.8 | 2.09 | | 0.05 | 0.08 | 99.4 | 0.56 | 0.00 | 0.00 | 98.4 | 1.53 | 0.03 | 0.05 |
| Go24 | | 99.5 | | 0.39 | | 0.00 | 0.15 | 98.9 | 0.86 | | 0.00 | 0.29 | 99.3 | 0.41 | 0.00 | 0.25 | 98.1 | 1.53 | 0.03 | 0.35 |
| Go25^a^ | | - | | - | | - | - | - | - | | - | - | - | - | - | - | - | - | - | - |
| Go26 | | 99.3 | | 0.64 | | 0.00 | 0.04 | 99.1 | 0.86 | | 0.00 | 0.03 | 95.1 | 4.84 | 0.00 | 0.09 | 98.2 | 1.63 | 0.03 | 0.14 |
| Go27 | | 99.2 | | 0.77 | | 0.01 | 0.03 | 97.8 | 2.17 | | 0.05 | 0.00 | 93.2 | 6.65 | 0.00 | 0.12 | 99.8 | 0.08 | 0.00 | 0.11 |
| Go28 | | 99.1 | | 0.78 | | 0.01 | 0.10 | 98.4 | 1.28 | | 0.05 | 0.21 | 93.3 | 6.61 | 0.00 | 0.12 | 98.1 | 1.58 | 0.03 | 0.25 |
| Go29 | | 99.5 | | 0.44 | | 0.00 | 0.04 | 99.1 | 0.86 | | 0.00 | 0.05 | 96.6 | 3.34 | 0.00 | 0.06 | 99.8 | 0.08 | 0.00 | 0.14 |
| Go30 | | 99.7 | | 0.30 | | 0.00 | 0.04 | 99.9 | 0.00 | | 0.00 | 0.05 | 98.0 | 1.81 | 0.03 | 0.12 | 98.3 | 1.53 | 0.03 | 0.11 |
| Go31 | | 99.5 | | 0.39 | | 0.02 | 0.07 | 99.8 | 0.03 | | 0.05 | 0.13 | 97.3 | 2.68 | 0.00 | 0.06 | 99.8 | 0.05 | 0.00 | 0.11 |
| Go32 | | 99.7 | | 0.30 | | 0.01 | 0.03 | 98.6 | 1.28 | | 0.05 | 0.03 | 99.6 | 0.34 | 0.00 | 0.03 | 98.4 | 1.53 | 0.03 | 0.03 |
| Go33 | | 99.1 | | 0.89 | | 0.01 | 0.02 | 97.8 | 2.17 | | 0.05 | 0.00 | 93.3 | 6.68 | 0.00 | 0.03 | 98.2 | 1.63 | 0.03 | 0.14 |
| Go34 | | 99.0 | | 0.84 | | 0.01 | 0.11 | 99.3 | 0.11 | | 0.05 | 0.53 | 93.2 | 6.65 | 0.00 | 0.12 | 98.2 | 1.66 | 0.03 | 0.08 |
| Go35 | | 99.4 | | 0.50 | | 0.01 | 0.05 | 97.8 | 2.11 | | 0.05 | 0.08 | 99.1 | 0.90 | 0.00 | 0.03 | 99.8 | 0.03 | 0.00 | 0.14 |
| Go36 | | 99.2 | | 0.75 | | 0.01 | 0.05 | 97.7 | 2.17 | | 0.05 | 0.08 | 93.3 | 6.65 | 0.00 | 0.06 | 99.8 | 0.08 | 0.00 | 0.08 |
| Go37 | | 99.2 | | 0.74 | | 0.01 | 0.02 | 97.8 | 2.17 | | 0.05 | 0.00 | 93.1 | 6.86 | 0.00 | 0.03 | 99.9 | 0.08 | 0.00 | 0.00 |
| Go38 | | 99.4 | | 0.58 | | 0.01 | 0.03 | 98.7 | 1.28 | | 0.05 | 0.00 | 99.5 | 0.53 | 0.00 | 0.00 | 99.9 | 0.05 | 0.00 | 0.03 |
| Go39 | | 99.1 | | 0.82 | | 0.02 | 0.05 | 98.6 | 1.36 | | 0.05 | 0.00 | 93.3 | 6.65 | 0.00 | 0.06 | 98.3 | 1.58 | 0.03 | 0.14 |
| Go40 | | 99.5 | | 0.49 | | 0.00 | 0.04 | 99.9 | 0.00 | | 0.00 | 0.08 | 99.5 | 0.37 | 0.00 | 0.09 | 99.8 | 0.05 | 0.00 | 0.11 |
|  | | **Total map (14.5 Gb)** | | | | | | **3B (829.5 Mb; carrying *Fhb1*)** | | | | | **5A (709.8 Mb; carrying *Fhb5*)** | | | | **6B (721 Mb; carrying *Fhb2*)** | | | |
|  | **A (%)** | | **B (%)** | | **H (%)** | | **U (%)** | **A (%)** | **B (%)** | **H (%)** | | **U (%)** | **A (%)** | **B (%)** | **H (%)** | **U (%)** | **A (%)** | **B (%)** | **H (%)** | **U (%)** |
| CDC Alsask (RP) | | 100.0 | | 0 | | 0 | 0 | 100.0 | 0 | | 0 | 0 | 100.0 | 0 | 0 | 0 | 100.0 | 0 | 0 | 0 |
| 04GC0139 (DP) | | 87.1 | | 12.91 | | 0.00 | 0.00 | 81.8 | 18.18 | | 0.00 | 0.00 | 83.3 | 16.68 | 0.00 | 0.00 | 77.6 | 22.40 | 0.00 | 0.00 |
| Alsask1 | | 98.0 | | 1.71 | | 0.05 | 0.21 | 94.9 | 4.87 | | 0.08 | 0.16 | 93.7 | 6.20 | 0.06 | 0.06 | 89.2 | 9.73 | 0.25 | 0.82 |
| Alsask2 | | 98.4 | | 1.55 | | 0.05 | 0.03 | 91.4 | 8.50 | | 0.11 | 0.00 | 98.2 | 1.75 | 0.03 | 0.03 | 98.2 | 1.74 | 0.03 | 0.05 |
| Alsask3 | | 98.6 | | 1.37 | | 0.03 | 0.01 | 92.7 | 7.30 | | 0.03 | 0.00 | 98.3 | 1.66 | 0.03 | 0.00 | 97.8 | 2.18 | 0.03 | 0.00 |
| Alsask4 | | 98.4 | | 1.54 | | 0.03 | 0.01 | 92.4 | 7.62 | | 0.03 | 0.00 | 98.1 | 1.85 | 0.03 | 0.00 | 97.6 | 2.40 | 0.03 | 0.00 |
| Alsask5 | | 98.4 | | 1.44 | | 0.04 | 0.07 | 91.4 | 7.78 | | 0.21 | 0.59 | 98.5 | 1.41 | 0.06 | 0.03 | 97.7 | 2.07 | 0.05 | 0.14 |
| Alsask6 | | 98.4 | | 1.56 | | 0.01 | 0.01 | 92.9 | 6.98 | | 0.08 | 0.00 | 89.4 | 10.55 | 0.03 | 0.00 | 98.7 | 1.23 | 0.03 | 0.00 |
| Alsask7 | | 98.7 | | 0.89 | | 0.06 | 0.32 | 96.5 | 2.30 | | 0.24 | 0.99 | 95.9 | 1.66 | 0.47 | 1.97 | 99.0 | 0.60 | 0.00 | 0.35 |
| Alsask8 | | 98.3 | | 1.58 | | 0.05 | 0.05 | 96.2 | 3.29 | | 0.13 | 0.37 | 98.0 | 1.63 | 0.16 | 0.25 | 88.0 | 11.83 | 0.08 | 0.05 |
| Alsask9 | | 98.2 | | 1.66 | | 0.04 | 0.10 | 95.6 | 4.09 | | 0.11 | 0.21 | 97.8 | 2.10 | 0.06 | 0.06 | 88.3 | 11.53 | 0.05 | 0.14 |
| Alsask10 | | 97.2 | | 2.46 | | 0.05 | 0.25 | 91.6 | 8.13 | | 0.05 | 0.24 | 90.1 | 9.70 | 0.06 | 0.16 | 87.0 | 12.29 | 0.11 | 0.60 |
| Alsask11 | | 97.3 | | 2.45 | | 0.07 | 0.19 | 91.4 | 8.42 | | 0.05 | 0.13 | 89.9 | 9.98 | 0.09 | 0.03 | 86.9 | 12.48 | 0.05 | 0.57 |
| Alsask12 | | 97.7 | | 1.75 | | 0.16 | 0.39 | 92.1 | 7.51 | | 0.13 | 0.21 | 90.4 | 9.26 | 0.16 | 0.19 | 89.9 | 2.43 | 2.34 | 5.29 |
| Alsask13 | | 97.7 | | 2.16 | | 0.04 | 0.12 | 91.7 | 8.10 | | 0.08 | 0.11 | 92.0 | 7.82 | 0.03 | 0.16 | 87.6 | 12.05 | 0.14 | 0.22 |
| Alsask14 | | 97.9 | | 2.05 | | 0.05 | 0.05 | 96.6 | 3.34 | | 0.03 | 0.00 | 98.0 | 1.85 | 0.06 | 0.09 | 87.7 | 12.18 | 0.08 | 0.05 |
| Alsask15 | | 97.9 | | 1.97 | | 0.04 | 0.06 | 92.0 | 7.75 | | 0.08 | 0.16 | 97.5 | 2.44 | 0.06 | 0.03 | 88.0 | 11.77 | 0.08 | 0.16 |
| Alsask16 | | 98.6 | | 1.33 | | 0.03 | 0.03 | 92.6 | 7.41 | | 0.03 | 0.00 | 98.3 | 1.66 | 0.03 | 0.03 | 97.7 | 2.18 | 0.03 | 0.05 |
| Alsask17 | | 97.6 | | 2.39 | | 0.04 | 0.01 | 91.7 | 8.26 | | 0.03 | 0.00 | 97.3 | 2.57 | 0.06 | 0.03 | 87.2 | 12.59 | 0.08 | 0.08 |
| Alsask18 | | 99.1 | | 0.89 | | 0.02 | 0.02 | 97.1 | 2.89 | | 0.00 | 0.00 | 93.8 | 6.17 | 0.00 | 0.00 | 99.3 | 0.65 | 0.03 | 0.05 |
| Alsask19 | | 98.8 | | 1.15 | | 0.02 | 0.01 | 97.8 | 2.17 | | 0.00 | 0.00 | 90.1 | 9.83 | 0.06 | 0.03 | 99.1 | 0.93 | 0.00 | 0.00 |
| Alsask20 | | 97.6 | | 2.41 | | 0.02 | 0.00 | 90.6 | 9.25 | | 0.11 | 0.00 | 89.5 | 10.42 | 0.06 | 0.00 | 87.7 | 12.24 | 0.08 | 0.00 |
| Alsask21 | | 97.2 | | 2.74 | | 0.04 | 0.03 | 91.2 | 8.69 | | 0.05 | 0.05 | 89.5 | 10.33 | 0.09 | 0.06 | 87.5 | 12.37 | 0.08 | 0.05 |
| Alsask22 | | 97.1 | | 2.88 | | 0.04 | 0.02 | 90.1 | 9.81 | | 0.08 | 0.00 | 88.3 | 11.58 | 0.09 | 0.00 | 87.2 | 12.62 | 0.05 | 0.08 |
| Alsask23 | | 98.8 | | 1.08 | | 0.05 | 0.04 | 96.8 | 3.07 | | 0.13 | 0.00 | 99.0 | 0.94 | 0.03 | 0.03 | 99.0 | 0.90 | 0.03 | 0.11 |
| Alsask24 | | 98.5 | | 1.47 | | 0.04 | 0.01 | 96.7 | 3.16 | | 0.11 | 0.00 | 90.0 | 9.92 | 0.06 | 0.00 | 98.8 | 1.17 | 0.00 | 0.00 |
| Alsask25 | | 97.5 | | 2.17 | | 0.11 | 0.20 | 96.7 | 2.91 | | 0.11 | 0.24 | 90.5 | 9.23 | 0.13 | 0.13 | 92.2 | 3.79 | 1.25 | 2.73 |
| Alsask26 | | 98.2 | | 1.72 | | 0.03 | 0.00 | 96.1 | 3.74 | | 0.11 | 0.00 | 90.5 | 9.39 | 0.06 | 0.00 | 98.7 | 1.25 | 0.00 | 0.00 |
| Alsask27 | | 98.1 | | 1.78 | | 0.03 | 0.09 | 89.9 | 9.92 | | 0.08 | 0.11 | 95.7 | 4.29 | 0.00 | 0.03 | 94.6 | 4.85 | 0.05 | 0.46 |
| Alsask28 | | 97.3 | | 2.61 | | 0.04 | 0.02 | 95.7 | 4.20 | | 0.08 | 0.00 | 88.3 | 11.61 | 0.09 | 0.00 | 89.6 | 10.28 | 0.05 | 0.03 |
| Alsask29 | | 98.6 | | 1.22 | | 0.08 | 0.11 | 96.4 | 2.99 | | 0.24 | 0.32 | 90.5 | 7.61 | 0.78 | 1.06 | 99.0 | 0.93 | 0.05 | 0.05 |
| Alsask30 | | 98.0 | | 1.85 | | 0.07 | 0.11 | 91.6 | 6.87 | | 0.51 | 1.07 | 89.1 | 10.61 | 0.06 | 0.19 | 98.3 | 1.50 | 0.14 | 0.05 |
| Alsask31 | | 98.2 | | 1.72 | | 0.04 | 0.01 | 96.1 | 3.74 | | 0.11 | 0.00 | 90.5 | 9.39 | 0.06 | 0.00 | 98.7 | 1.25 | 0.00 | 0.00 |
| Alsask32 | | 98.0 | | 1.97 | | 0.04 | 0.02 | 96.7 | 3.16 | | 0.05 | 0.05 | 89.1 | 10.70 | 0.09 | 0.06 | 90.9 | 9.02 | 0.08 | 0.00 |

**Fig. S1.** **Polymorphism in CDC Go and CDC Alsask near-isogenic lines (NILs).** Number of polymorphic (among NILs and recurrent parents only) single nucleotide polymorphism (SNP) markers in CDC Alsask and CDC Go NILs segregating for *Fhb1* (3B), *Fhb2* (6B), and *Fhb5* (5A).


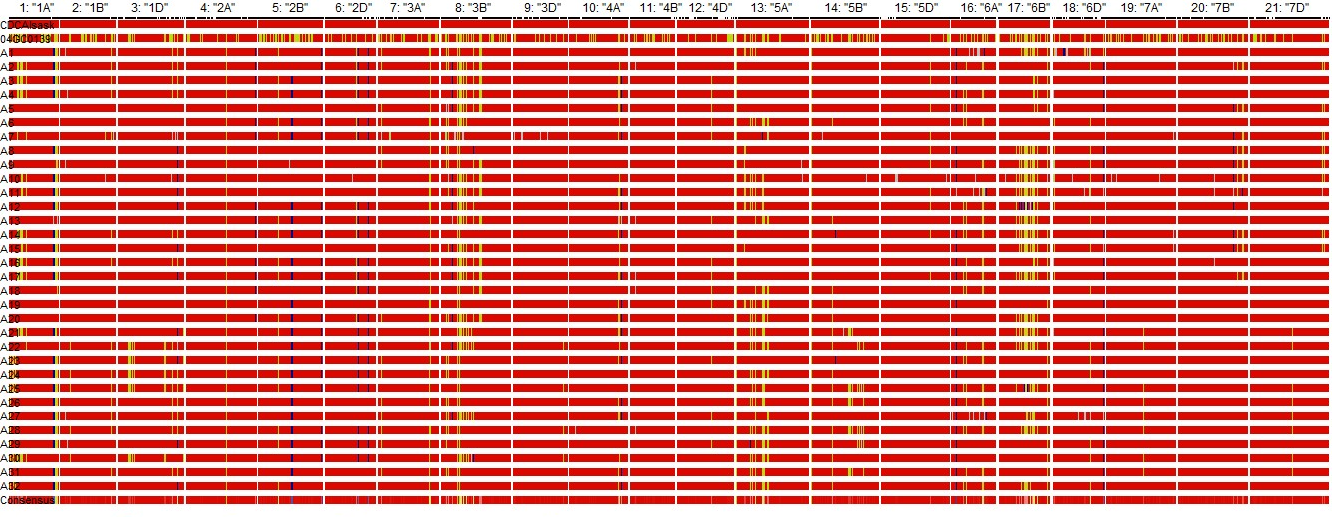


**Fig. S2.** **Polymorphism in CDC Alsask near-isogenic lines on Chromosomes other than 3B, 5A, 6B.** Graphical presentation of introgressed segments in all chromosomes, except 3BS, 6BS, and 5AS, from 04GC0139 (resistance donor parent, yellow segments) into CDC Alsask (red segments) near-isogenic lines. Each bar represents a genotype. The grey and blue segments indicate unknown and heterozygous alleles, respectively.


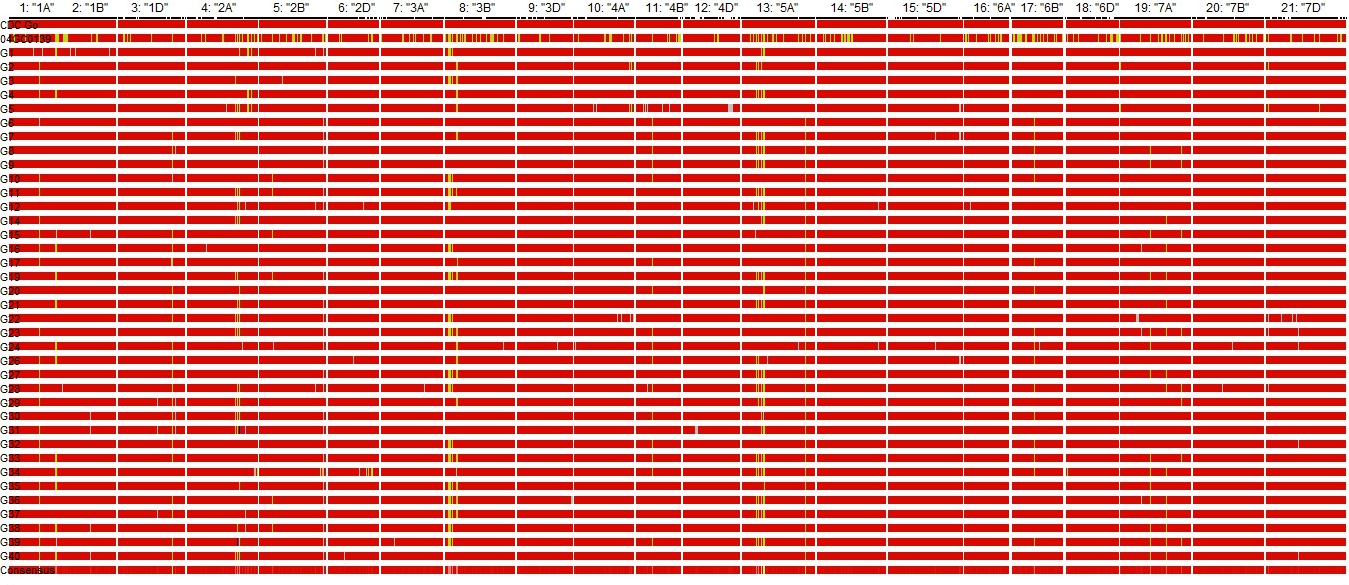


**Fig. S3.** **Polymorphism in CDC Go near-isogenic lines on Chromosomes other than 3B, 5A, 6B.** Graphical presentation of introgressed segments in all chromosomes, except 3BS, 6BS, and 5AS, from 04GC0139 (resistance donor parent, yellow segments) into CDC Go (red segments) near-isogenic lines. Each bar represents a genotype. The grey and blue segments indicate unknown and heterozygous alleles, respectively.

**
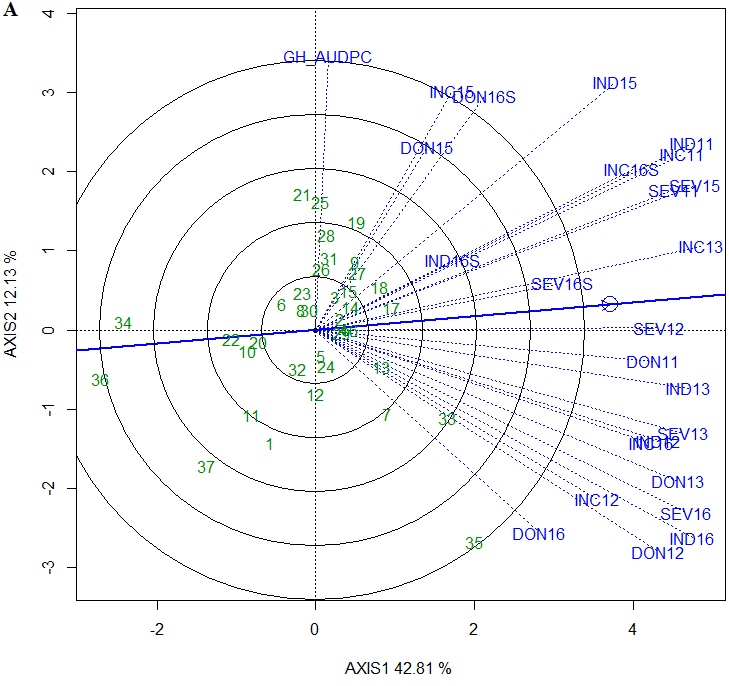

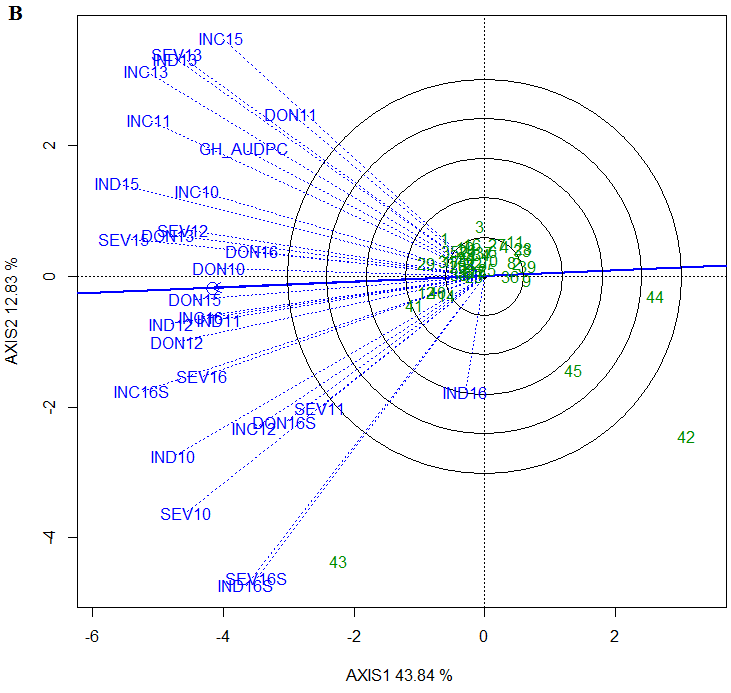
**

**Fig. S4. GGE Biplots for CDC Go and CDC Alsask near-isogenic lines (NILs).** Genotype and genotype by environment (GGE) interaction plot showing the relationship among genotypes, environments and their interaction for: (A) CDC Alsask, and (B) CDC Go NILs. Numbers in the green indicate NIL entries and vectors (dotted blue lines) are unique to given environment (blue labels for vectors). The solid blue line passing through the origin of the plot is the ‘Average Environment Axis’ indicating the most ideal and discriminating environment. The axes of the plot indicate standard deviation for phenotype (proportional to length of environment vector). The phenotypic variation explained by both axes is indicated next to the axes labels. Here: INC, SEV, IND, DON, GH_AUDPC represent field incidence, field severity, field index, field deoxynivalenol accumulation, and greenhouse area under disease progress curve.
